# Enzyme Technology in the Food Industry: Molecular Mechanisms, Applications, and Sustainable Innovations

**DOI:** 10.1002/fsn3.70927

**Published:** 2025-09-17

**Authors:** Fahima Siddikey, Md Istiakh Jahan, Hormoni, Md Toufik Hasan, Nusrat Jerin Nishi, S. M. Kamrul Hasan, Nahidur Rahman, Md. Azmain Al Faik, Mohammad Afzal Hossain

**Affiliations:** ^1^ Department of Food Engineering and Tea Technology Shahjalal University of Science and Technology Sylhet Bangladesh; ^2^ Department of Food Processing and Preservation Hajee Mohammad Danesh Science and Technology University Dinajpur Bangladesh; ^3^ Department of Food Processing and Engineering Chattogram Veterinary and Animal Sciences University Chattogram Bangladesh; ^4^ Department of Food Engineering and Nutrition Science State University of Bangladesh Dhaka Bangladesh

**Keywords:** enzyme production, mechanisms, microbial enzyme, plant enzyme, solid‐state fermentation, submerged fermentation

## Abstract

Enzymes are protein‐based biocatalysts that speed up biochemical reactions without affecting equilibrium, with microbial sources being the most efficient and cost‐effective for large‐scale production. Their dual roles as processing aids and inhibitors of spoilage make understanding their structure, function, and specific activity vital for both in vitro applications and food product development. While the development of recombinant DNA technology and the advancements achieved by microbes in food applications are more economically and practically advantageous, certain of these enzymes are plentiful enough in their natural sources to allow for large‐scale synthesis. Enzymes find extensive use across many domains, including but not limited to agriculture, environment, paper and pulp, leather tanning, chemicals and pharmaceuticals, detergents, food and drink, and so on. In this review, we have focused on the molecular mechanisms of enzyme activity and the enzyme manufacturing process within the food industry. We also dedicate a section to various enzymes used in this sector, including amylase, lipase, proteases, cellulase, lactase, glucose oxidase, glucose isomerase, invertase, catalase, and rennet. Additionally, we have presented case studies showcasing the successful implementation of sustainable enzyme practices across different food sectors, highlighting both practical benefits and challenges. The review concludes with an outlook on future trends and research directions aimed at achieving greater safety and sustainability in enzyme technology within the food industry.

## Graphical abstract

This review explores the role of microbial and plant enzymes in food processing, highlighting advancements in molecular engineering and sustainable production from underutilized agro‐industrial waste. It focuses on key enzymes such as amylase, lipase, protease, and lactase, discussing their applications, advantages, and challenges in enhancing product quality, efficiency, and sustainability. Additionally, the paper identifies emerging trends, technological innovations, and future directions for developing customized and robust enzymes tailored to meet the diverse needs of the food industry.

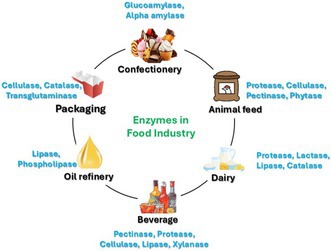

## 1 Introduction

Enzymes, also known as biological catalysts, play a key role in enhancing biochemical reactions in living systems. They have the capacity to be extracted from cells, thereby facilitating a number of commercially important processes (Robinson 2015). By binding to substrates preferentially and causing morphological modifications to the substrate molecules, they can promote the occurrence of the reaction as well as increase the rate of reaction (Ackaah‐Gyasi et al. 2015). The literature has provided characterizations of a wide variety of enzymes derived from diverse sources. Traditionally, enzymes are obtained from a variety of sources, including microorganisms, animals, and plants. Nevertheless, the commercial synthesis of these enzymes is often microbiological in origin, derived from bacteria, yeast, and fungus, with the majority produced via submerged fermentation (SMF) or solid‐state fermentation (SSF) (Londoño‐Hernandez et al. 2020). Over 50% of the enzymes used in industrial settings come from fungi, including molds and yeasts. Notable fungi for enzyme synthesis are *Aspergillus*, *Penicillium*, *Candida*, *Saccharomyces*, and *Mucor* (El‐Gendi et al. 2021). Meanwhile, another 30% of the enzymes are derived from bacteria such as *Streptomyces*, *Lactobacilli*, *Klebsiella*, and *Bacillus* (Liu and Kokare 2023). Around 8% of industrial enzymes, primarily sourced from animals like cow and pig pancreas, liver, and stomach, are predominantly utilized in food and pharmaceutical applications, while enzymes from other animals, such as fish, are also sought after for their adaptability to a wider range of environmental conditions (Aminlari 2022). About 4% of industrial enzymes used in industry come from edible, non‐toxic plant species. In general, microbial enzymes, due to their lower production costs, ease of quantity control, and consistent availability in predictable amounts, are utilized in the highest quantities in industry (Singh, Kundu, et al. 2019).

**FIGURE 1:**
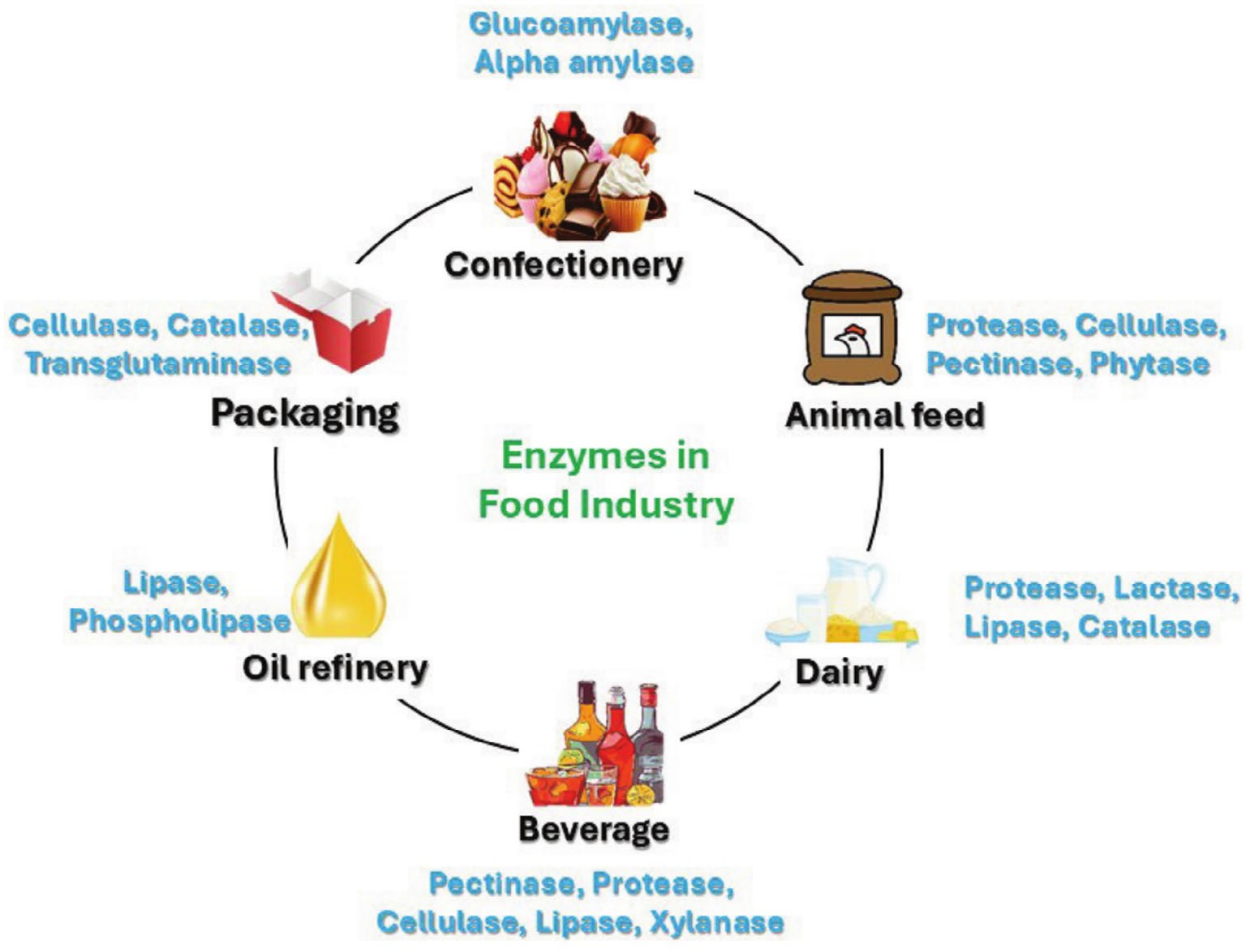
Schematic representation of enzyme usage in different sectors of the food industry.

In many industrial processes, including pharmaceuticals, polymers, detergents, paper, textiles, cosmetics, and leather processing, enzymes are utilized. The food industry extensively employs enzymes in numerous applications, including dairy products, confections, and packaging (Figure 1). Amylase, lipase, protease, isomerase, peroxidase, polyphenol oxidase, and oxidoreductase are some of the important food‐processing enzymes widely used in fermented products, dairy products, spices, bakery products, food additives, starch processing, wine, and the fermented products, wine, and beverage industries (Hbaieb et al. 2017). In addition, enzymes improve stability and are utilized in the production of vegetable juice, the extension of the shelf life of vegetables and fruits, and the reduction of nutritional loss (Toushik et al. 2017). The global food enzymes market had a valuation of $2058 million in 2020 and is projected to reach USD 4194 million by 2032, with a revenue compound annual growth rate (CAGR) of 6.1% (Yang et al. 2023).

However, to maintain a continuous right direction and unlock its huge scope for future growth, it is necessary to adopt modern production methods that will overcome the shortcomings of traditional production processes. At present, progress in the field of molecular biology has provided scientists the ability to easily alter enzymes' DNA to improve their properties and reduce expenses (Singh, Kundu, et al. 2019; Singh, Singh, and Sachan 2019; Singh, Singh, and Pandey 2019). The discovery and modification of new food enzymes is essential for improving food processing and enhancing product quality. Food enzymes with certain functions have previously been obtained by researchers by screening microorganisms and purifying and identifying the proteins; however, this process is inefficient and requires more investigation (Wiltschi et al. 2020).

Moreover, there is a need for further exploration into the effective utilization of enzymes in the food industry. There are still many obstacles in the way of the manufacturing, application, and study of food enzymes; in order to make significant progress in these areas, a thorough investigation and resolution of these problems are required. Despite advancements in enzyme biotechnology, there is still a significant gap in the exploration of waste‐derived sources for microbial enzyme production, which could offer a dual benefit of waste reduction and resource utilization. Furthermore, most current reviews lack comprehensive coverage of both the technological advances in enzyme design and the integration of sustainable food production methods. Although enzymes have long been used in the food industry, their broader application faces several critical limitations, including sensitivity to processing conditions, narrow substrate specificity, and high production costs. Additionally, sustainability concerns and consumer demand for clean‐label products are pushing the industry to innovate beyond traditional enzyme uses. This review aims to explore how current advances in enzyme technology—including molecular engineering, biotechnological tools, and sustainable production of microbial enzymes from underutilized agro‐industrial wastes—are addressing these challenges. The novelty of this review lies in its focus on underutilized agro‐industrial waste as a sustainable substrate for microbial enzyme production, coupled with a detailed evaluation of enzyme applications in the food industry and their potential for enhancing food‐processing efficiency. By linking traditional knowledge with emerging innovations, the review identifies key trends, gaps, and future directions that can shape the next generation of enzyme‐based solutions in food processing and functional product development.

## 2 Molecular Mechanisms of Enzymes in the Food Industry

Before delving into the advances and applications of enzymes in the food industry, it is crucial to first understand the molecular mechanisms underlying enzyme function. Enzymes, as biological catalysts, play a crucial role in driving biochemical reactions such as fermentation, flavor development, and texture modification in food products. Therefore, understanding how enzymes interact with their substrates, the catalytic mechanisms they utilize, and how environmental factors influence their activity is essential. Such insights can help optimize food‐processing techniques within the industry. Moreover, this knowledge contributes to more sustainable production by reducing energy consumption, minimizing waste, and decreasing the reliance on chemical additives in food processing.

### 2.1 Enzyme–Substrate Interactions

**FIGURE 2:**
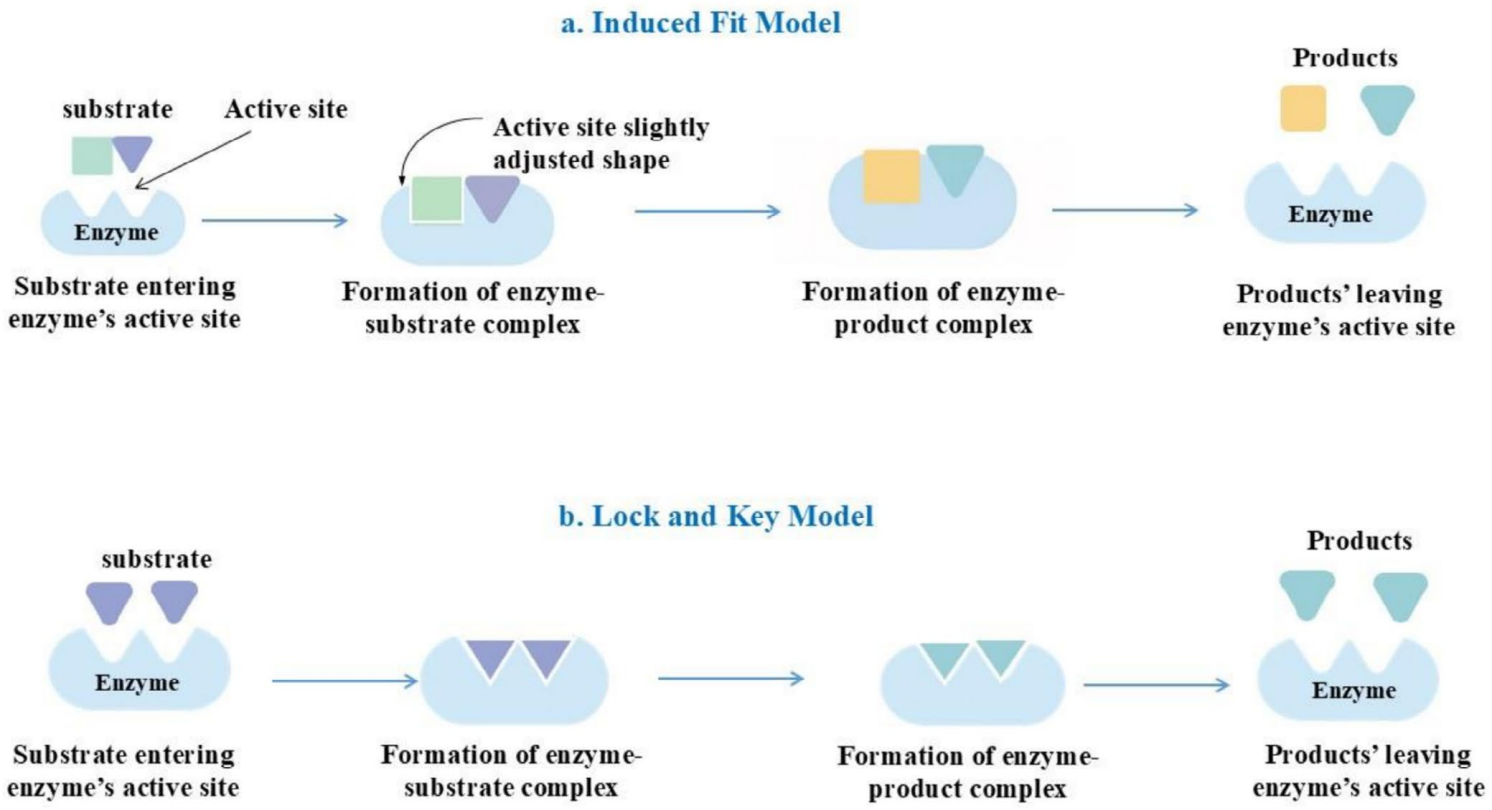
Enzyme–substrate interaction through (a) induced‐fit model and (b) lock‐and‐key model.

Enzymes exhibit high specificity, which is a hallmark of their function. This specificity refers to an enzyme's ability to selectively bind to and catalyze a reaction with a specific substrate. The degree of specificity is largely determined by the flexibility of the enzyme's active site (Heinemann et al. 2021). The active site is a specific region of an enzyme that interacts with the substrate. Depending on its structural flexibility, substrate recognition can follow either the lock‐and‐key mechanism or the induced‐fit mechanism (Figure 2).

In the lock‐and‐key model, the active site of the enzyme is rigid, and it binds to the substrate in a manner similar to a key fitting into a lock. The enzyme and substrate fit together perfectly, allowing the enzyme to catalyze the reaction without changing its structure significantly. Pectinase serves as an excellent example of this model, where it binds to pectin during juice preparation and processing (Tatta et al. 2022). In contrast, the induced‐fit model involves a more dynamic interaction. Here, the enzyme's active site undergoes a conformational change upon substrate binding, adjusting to better accommodate the substrate. This flexibility enhances the binding affinity and catalytic efficiency. A prime example of the induced‐fit model is lactase, which binds to lactose and undergoes conformational changes to break it down into simpler sugars (Agrawal 2024).

The activity of an enzyme's active site can be regulated by cofactors and coenzymes. Cofactors are typically inorganic molecules, often metal ions like magnesium or zinc, that assist in the enzyme's catalytic process. Coenzymes, on the other hand, are non‐protein organic molecules that also play a significant role in improving enzyme activity. Both cofactors and coenzymes facilitate the proper functioning of the enzyme by aiding in substrate binding or stabilizing the transition state, thus increasing the enzyme's efficiency.

### 2.2 Catalytic Mechanisms

Enzyme catalysis helps to lower the activation energy required for a chemical reaction to proceed. The higher the activation energy, the slower the reaction rate. By reducing the activation energy, enzymes accelerate the reaction process. Enzymes achieve this by employing several catalytic strategies, including covalent catalysis, acid–base catalysis, and metal ion catalysis.

In acid–base catalysis, enzymes such as amylase facilitate the reaction by donating a proton (H^+^) to the starch substrate. This proton donation lowers the activation energy needed for the hydrolysis reaction to occur, thereby accelerating the cleavage of glycosidic bonds and converting starch into simpler sugars (Likhtenshtein 2025). Covalent catalysis is exemplified by lipase, which hydrolyzes ester bonds in triglycerides, releasing fatty acids. In this mechanism, the enzyme forms a temporary covalent bond with the substrate, facilitating the reaction until the product is released (Ni et al. 2025). Metal ion catalysis involves metal ions, such as zinc and magnesium, present in the enzyme's active site. For instance, proteases such as trypsin and chymotrypsin utilize metal ions to facilitate peptide bond cleavage during protein hydrolysis (Jamal et al. 2025). Metal ions help stabilize the transition state, thereby reducing the activation energy required for the reaction.

### 2.3 Conformational Changes and Regulation

**FIGURE 3:**
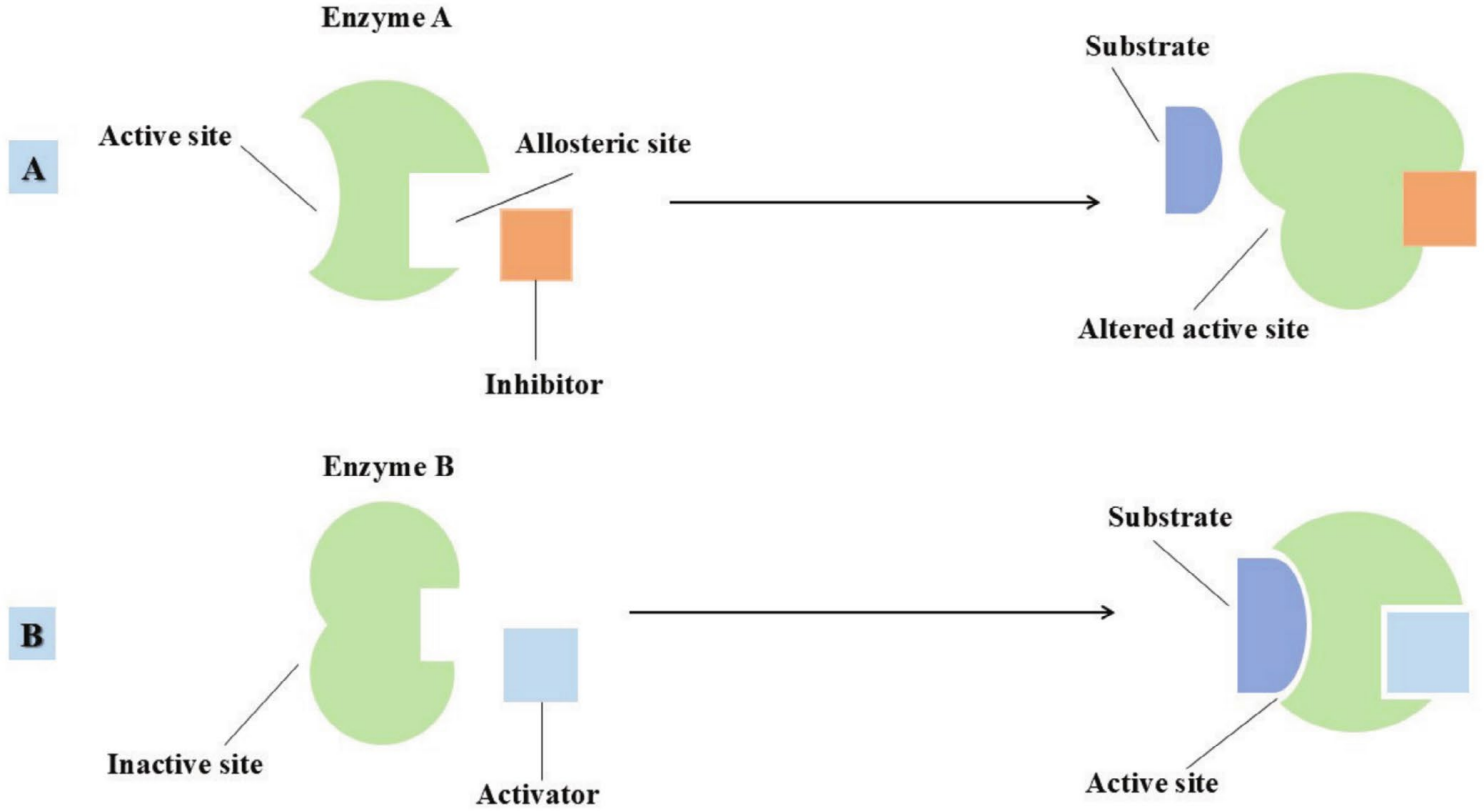
Enzyme conformational change and regulation through allosteric inhibition (A), allosteric activator (B).

When an enzyme binds with its substrate, it undergoes a conformational change that enhances its catalytic efficiency and optimizes the active site for the reaction. Along with the active site, enzymes may also possess an allosteric site, where molecules can bind to regulate the enzyme's activity. Binding at this site induces a conformational change in the enzyme, which can either increase or decrease its activity. The molecules that bind to allosteric sites are known as allosteric regulators (Figure 3). For example, in glucose syrup production, the activity of glucose‐6‐dehydrogenase is increased when NADP+ binds to an allosteric site, facilitating the production of glucose‐6‐phosphate (Yoshida and Lin 1973).

Another important regulatory mechanism is feedback inhibition. In this process, the final product of a metabolic pathway binds to and suppresses the activity of an upstream enzyme, thereby regulating the pathway and avoiding excess product formation. For instance, in citric acid production, the end product, citric acid, inhibits the enzyme aconitase, thus regulating and preventing the excessive accumulation of citric acid (Ranjan and Dubey 2023).

### 2.4 Enzyme Engineering for Improved Mechanisms

Over the years, a variety of molecular biology techniques have been used to enhance substrate specificity, enzyme activity, and stability. These techniques include site‐directed mutagenesis, directed evolution, de novo enzyme design, and metagenomics. Through the application of these methods, scientists successfully engineered enzymes with significantly enhanced properties. For instance, enzymes that traditionally function under acidic conditions can now operate optimally in alkaline environments, and enzymes that previously performed well only at room temperature can now function effectively across a broader range of temperatures, both low and high. In this section, we will briefly discuss a few of these groundbreaking techniques, which have made such advancements possible and paved the way for enzymes with enhanced capabilities.

Site‐directed mutagenesis is a molecular biology technique that induces specific mutations (such as substitutions, insertions, or deletions) at designated locations in a gene or DNA sequence. These alterations to the nucleotide sequence can directly impact the enzyme's function, structure, and overall biological activity. Researchers have leveraged this technique to engineer enzymes, particularly by improving their active sites, which enhances enzyme specificity, activity, and stability. Several studies have successfully applied site‐directed mutagenesis to improve catalytic efficiency and enzymatic performance. For instance, the technique has been used to enhance the activity of glucoamylase from *Talaromyces leycettanus* JCM12802 (Tong et al. 2021), β‐galactosidase from *Streptococcus thermophilus* (Zhao et al. 2022), and lipase from *Bacillus licheniformis* (Almansoori et al. 2025).

**FIGURE 4:**
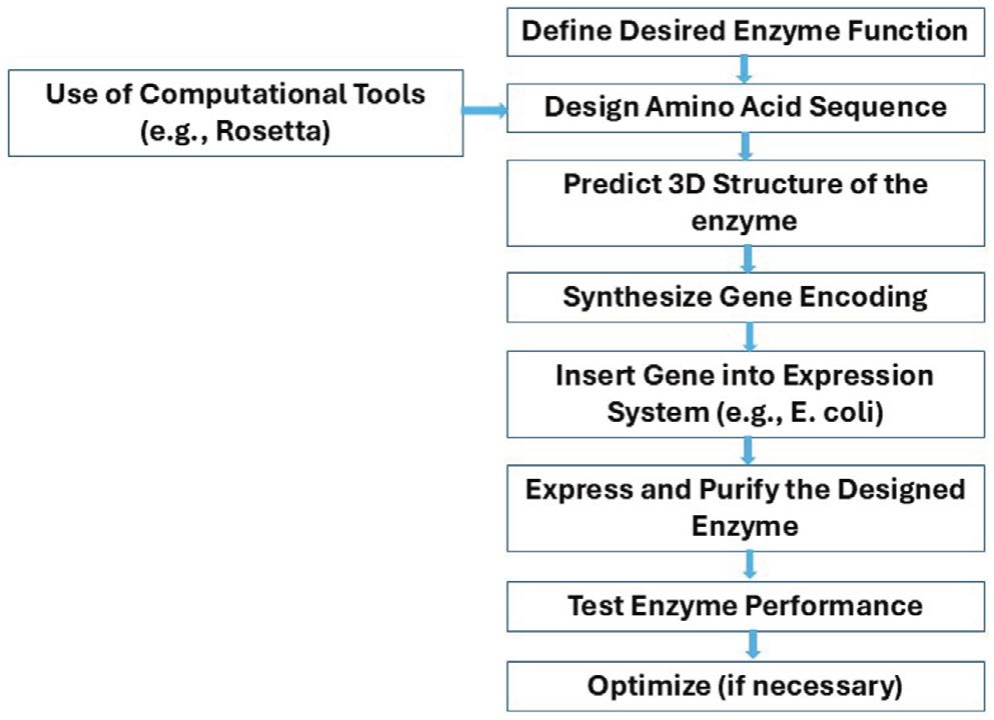
Flow diagram describing the steps of de novo enzyme design process.

*De novo* enzyme design is an advanced biotechnological approach that involves creating an entirely new enzyme with a specific desired function and amino acid sequence. This method typically involves the use of computational tools and requires a thorough understanding of protein folding, enzyme catalysis, and biochemical pathways (Korendovych and DeGrado, 2020; Kortemme 2024). At the outset of the design process, researchers define the specific function they aim to achieve. Using computational tools such as Rosetta or other protein modeling software, they design an amino acid sequence that will form the enzyme's active site, thereby facilitating the desired function. The next step is to predict the three‐dimensional (3‐D) structure of the enzyme to ensure the active site is optimized for efficient catalysis. Following this, the designed amino acid sequence is converted into a synthetic gene, which is then inserted into an expression system (e.g., *Escherichia coli* ) for protein production. Once the enzyme is synthesized and produced, its performance is tested. If the expected activity is not fully achieved, further optimization is performed to enhance its function (Figure 4) (Korendovych and DeGrado 2020).

In recent years, metagenomics, the branch of genomics dedicated to analyzing genetic material directly extracted from environmental samples, has greatly advanced the discovery of new enzymes. Researchers have been screening microbial species and strains from extreme environments, identifying microbes capable of producing novel enzymes with enhanced properties that function optimally under harsh conditions. This has opened new possibilities for food‐processing applications. For example, a cold‐adapted glucose oxidase from *Cladosporium neopsychrotolerans* SL16 demonstrates optimal activity at 20°C (Ge et al. 2020), whereas the traditional glucose oxidase has an optimal temperature of 40°C (Zia et al. 2007). Additionally, a thermostable lipase isolated from *Brevibacillus thermoruber* strain 7 exhibits optimal activity at 55°C and pH 7.5 (Atanasova et al. 2023). In contrast, lipase from *Pseudomonas gessardii* has been reported to show optimum activity at pH 5.0 and 37°C (Ramani et al. 2010).

For example, in the production of high‐fructose corn syrup, glucoamylase from *Aspergillus niger* is modified to improve its stability at high temperatures and an optimal pH of around 5.0. Specific amino acid residues, such as glutamine and glutamic acid, were altered to improve the enzyme's stability and activity under these conditions. These modifications increased the enzyme's efficiency in converting starch into glucose, improving the yield of high‐fructose corn syrup in industrial applications. These engineered enzymes are tailored to function more effectively under specific conditions, such as elevated temperatures or altered pH, improving process efficiency and product yield (Pavezzi et al. 2008).

Recent breakthroughs in protein engineering and molecular biology have significantly advanced the ability to design and optimize enzymes with tailored properties, such as specificity, enhanced stability, and catalytic efficiency (Ndochinwa et al. 2024). Recombinant DNA technology has enabled the production of thermophilic enzymes by inserting specific genes like thermophilic amylase from *Bacillus stearothermophilus* (amyA gene) and lipase from *Thermomyces lanuginosus* (lipA gene) into bacterial strains such as *E. coli* (Ndochinwa et al. 2024). These engineered enzymes show high activity even at elevated temperatures, making them invaluable in food‐processing industries requiring heat, such as brewing, baking, and dairy processing (Cheng et al. 2021). This ability to withstand high temperatures and remain functional under extreme conditions allows for improved efficiency in industrial applications.

In addition to enhancing enzyme activity through genetic modifications, protein engineering has been used to further optimize enzyme properties. Directed evolution and rational design are key techniques employed to achieve this. In directed evolution, random mutations are introduced into the gene of interest (e.g., amylase from *Aspergillus niger* (amy1 gene)), followed by screening to identify variants with improved properties, such as increased substrate specificity or greater thermal stability (Patel, Dong, Chen, Pandey, and Singhania 2023). Rational design uses knowledge of enzyme structure to make specific alterations at key amino acid sites, as seen in lipase from *Candida antarctica* (CALB gene), which has been engineered to improve its efficiency in transesterification reactions for biodiesel production and flavor enhancement in food applications (Chen et al. 2022).

Furthermore, enzyme immobilization techniques have become essential for improving enzyme stability, reusability, and cost‐effectiveness (Maghraby et al. 2023). By attaching enzymes to solid supports (e.g., ceramics, polymers, nanoparticles), immobilization prevents enzyme loss during reactions and allows for easier recovery and reuse. This approach is particularly useful in continuous food‐processing systems where enzymes can be employed over extended periods without significant loss of activity. Recent advancements in nanomaterial‐based immobilization have resulted in enzymes that exhibit enhanced resistance to extreme conditions, such as organic solvents and high temperatures, making them ideal for complex food production processes (Miah, Chy, Ahmed, et al. 2025).

## 3 Production of Commercial Enzyme

The production of industrial enzymes for commercial use typically involves microorganisms sourced from various species, including animals, plants, and bacteria, which are cultured using either SSF or SMF. Notably, the vast majority of enzyme classes found in food products are produced using fungal hosts.

### 3.1 Microbial Enzyme

At present, approximately 200 different microbial enzymes out of the total of 4000 known enzymes are being utilized for commercial purposes (Liu et al. 2023). However, only 20 enzymes are manufactured on a large scale in the industrial sector.

Glucose oxidase, an important oxidoreductase enzyme, is industrially derived from *Penicillium glaucum* and *Aspergillus niger* using a technique that is SSF. It has broad uses in food processing for its roles in antioxidation, preservation, and product stabilization. It also significantly influences the development of color, taste, and texture, as well as extending the shelf life of food items (Khurshid et al. 2011). Bharwani et al. (2024) devised the Milk Adulteration Testing and Analysis kit, which utilizes immobilized glucose oxidase enzyme to quickly detect cow milk that has been adulterated with urea and glucose.

Lipases, which catalyze the hydrolysis of ester bonds in lipid substrates, are key contributors in the baking, dairy, juice processing, winemaking, and brewing sectors. Microorganisms like *Pseudomonas aeruginosa* , *Staphylococcus aureus* , and *Bacillus subtilis* are regarded as excellent microbial sources of lipase enzymes. Lipases have various commercial applications in the food industries, including the breakdown of milk lipids, enhancing the flavor of cheese, reducing bitterness, and preventing rancidity. Lipases can produce a wide range of flavor profiles when they interact with milk fat. For instance, Hamdy et al. (2017) used non‐fat milk and lipolyzed cream using lipase sourced from *Rhizomucor miehei* to create Ras cheese curd, which was subsequently used to produce a Ras cheese flavor concentrate.

Microbial enzymes are considered superior to animal‐ and plant‐based enzymes in a number of ways. When compared to their plant and animal counterparts, microbial enzymes are remarkably stable and active, which is an important feature that greatly increases their effectiveness in a variety of applications (Raveendran et al. 2018). The biochemical diversity and gene manipulation capabilities of enzymes derived from microorganisms enable high yields and facilitate easy optimization. Due to their variety and abundance, microbes are a valuable resource for discovering microbial enzymes through innovative techniques like genome mining, metagenome screening, and extremophile exploration (Adrio and Demain 2014).

### 3.2 Plant Enzyme

Plants are not typically used for enzyme production because their growth can vary with seasons and geographical locations. Additionally, since most plant‐derived enzymes are intracellular, the purification process is more time‐consuming (Aminlari 2022). Nevertheless, a wide range of enzymes has traditionally been produced from plant sources. Several plant tissues have been utilized to extract and purify a variety of enzymes, including lipase, ascorbate oxidase, bromelain, phytase, sucrose, nitrate reductase, urease, hydroxyl nitrile lyase, protease, and papain. Among these, bromelain is commonly extracted from the pineapple ( *Ananas comosus* (L.) Merr.) stem, while papain is obtained from the papaya plant ( *Carica papaya* ), and ficin is derived from fig latex (Gagaoua et al. 2014). The manufacturing of syrups, bakery goods, alcoholic beverages, and dairy products has been significantly influenced by these enzymes. Table 1 enlists the enzyme sources, their applications, and their contributions to the food industry.

**TABLE 1:** An overview of enzymes used in different sectors of the food industry.

Enzyme	Microbial source	Industries	Objectives	Process parameters	Key findings	References
α‐Amylase	*Bacillus* sp., *Aspergillus* sp.	Baking, Beverage, Starch	To control volume and crumb structure of bread, convert starch to glucose syrup, decrease viscosity and rise maltose and glucose content, and improve thermostability	TS‐αA concentrations of 0.0% to 1.6%, and MS‐αA concentrations of 0.0% to 2.0% were used for dough preparation Cooking qualities and sensory properties were evaluated	Thermostable (TS‐αA) and mesophilic (MS‐αA) α‐amylases enzymes modified wheat starch into water‐soluble oligosaccharides and dextrin Promoted starch gelatinization at appropriate concentrations. 1.6% MS‐αA resulted in extruded noodles with an improved porous structure, rehydration and palatability. TS‐αA and MS‐αA concentrations of 0.8% and 1.6% improved firmness, elasticity and chewiness qualities of cooked noodles	Li et al. (2019)
Acetolactate Decarboxylase	*Bacillus subtilis*	Beverage	To improve taste and off‐flavor prevention in beer		Immobilized α‐acetolactate decarboxylase (ALDC) effectively converted α‐acetolactate to acetoin in beer After 12 cycles, it maintained 80% of its original activity, highlighting strong operational stability. Represented the most stable ALDC formulation for beer applications, successfully prevented off‐flavor in the product.	Costa et al. (2022)
Aminopeptidase	*Lactobacillus brevis* , *L. plantarum*	Dairy, Beverage	To faster cheese ripening and improve sensory properties	Addition:Aminopeptidase: 2 U/g of protein, orA‐CM: 0.02 g/kg of raw milk. Incubation: 32°C for 30 min. Rennet addition: 0.05 g/L and coagulation: 32°C for 1 h. Dry salting: 30 g/kg (wt/wt of curds). Ripening: 13°C to shorten maturation time.	A‐CM cheeses had 15× higher levels of 2/3‐methylbutanal and ethyl butyrate compared to controls. A‐CM cheeses showed the highest proteolysis levels, surpassing control and aminopeptidase‐added cheeses. No significant differences in hardness or springiness across cheese groups. Volatile compounds like 3‐methylbutanal and ethyl butyrate were significantly higher in A‐CM cheeses. A‐CM cheeses received the highest taste and aroma ratings.	Li et al. (2024)
Amyloglucosidase	*Aspergillus niger*	Baking, Starch	To improve bread quality	Amount of AMG (Amyloglucosidase): 4 U/g starch, 95°C for 10 min. Buffer Solutions: 5 g starch in 25 mL of 20 mM NaH_2_PO_4_ (pH 6.0) or sodium acetate buffer (pH 4.0). Incubation: Shaking at 50 rpm, 50°C for 24 h. Enzyme Inactivation: Boiled for 10 min, freeze‐dried	Gelatinization Properties: AMG‐treated starch showed higher onset temperature (67.48°C) and lower ΔH, indicating less thermal energy needed. AMG‐treated starch represented higher maximum viscosity, indicating improved cooking stability and viscosity retention A higher reaction rate (0.054 mg/100 g/s) compared to the control (0.036 mg/100 g/s), indicating greater susceptibility to enzymatic hydrolysis. A lower digestibility constant (k) (0.017 min^−1^) than the control (0.027 min^−1^), suggesting slower diffusion of amylase into the starch granules.	Dura et al. (2014)
β‐galactosidase	*Staphylococcus* sp., *Kluyveromyces* sp., *Escherichia coli*	Beverage, Baking	Able to hydrolysis of lactose and production of D‐galactose	2.00 mL crude cell extract of *P. haloplanktis* β‐galactosidase (1 mg enzyme). 20.00 mL model lactose solution 20.00 mL whey permeate with 34% dry matter. pH 7.0 Incubation for hydrolysis:Temperature: 23°C, Time: 24 h. Enzyme inactivation by boiling for 3 min.	Highest galactose concentration: 59.7 g/L Lactose conversion rate was 96% Hydrolysis efficiency at pH 7.0 and 8.0: Similar efficiencies achieved Stability of β‐galactosidase maintained over 90% for several cycles Optimal incubation temperature was 23°C Galacto‐Oligosaccharides production: Higher at 23°C compared to 15°C Inhibition by reaction products: glucose and galactose inhibit enzyme activity	Van De Voorde et al. (2014)
Cellulase	*Aspergillus niger*, *Trichoderma atroviride*, *Bacillus* sp.	Beverage	Juice extractions	Enzyme Concentration: 50 U Pectinase and 50 U Cellulase PH: 4 Incubated at 50°C for 20–300 min, enzyme inactivated at 90°C for 5 min. Juice is processed as per traditional extraction.	Highest total soluble solids recovery (72.37 g/100 g fresh basis) & lowest turbidity (186.45 NTU) at Pulp: water ratio 1:3, 50 U Pectinase, 5 U Cellulase, 120 min at 50°C. Enzyme‐treated syrup had higher Consumer Preference. Free from aerobes, molds, coliforms, Enterobacteriaceae; stable for 5 months.	Abbès et al. (2011)
Glucose isomerase	*Streptomyces murinus* , *Corynebacterium* sp., *Penicillium chrysogenum*	Starch, Organic synthesis	To produce high‐fructose corn syrup	Immobilization of GI Cells: flocculated with PEI, immobilized with GA, filtered, washed, dried. Amount of cells 0.03 g in 10 mL phosphate buffer. Temp: 0°C–60°C; Time: 30–180 min.	Immobilized glucose isomerase was active above 90% in pH 6.0–9.0 at 85°C–95°C More than 55% fructose concentration was obtained. 85% of the initial activity was still retained after reused for 15 days. Potential biocatalyst for HFCS‐55 production.	Jin et al. (2017)
Glucose oxidase	*Aspergillus niger*	Baking, Beverage, Polymer	To remove oxygen from beer, Dough strengthening, Polymerization of anilines	Cutted pieces of carps were randomly assigned to four treatments‐1.25% SBS, 0.5% Vitamin C, 1 U/mL GOxP5 (prepared from 12.5 U/mL), sterile water (control) Dipping Time: 10 min for all treatments. Polythene packaging, sealed, stored at 4°C for 10 days & analyses conducted every 2 days	GOxP5 treatment maintained a sensory score of 5 on day 10. GOxP5 showed a significant decline in sensory quality compared to the control but outperformed SBS and Vc treatments. GOxP5 had a significantly lower TBC (6.58 log CFU/g) than the control group (8.04 log CFU/g) on day 10, indicating better microbial quality. GOxP5 had a TVB‐N value of 25.2 mg/100 g on day 6, below the acceptable limit of 30 mg/100 g, while the control exceeded this at 35.85 mg/100 g.	Yuan et al. (2020)
Hemicellulase	*Trichoderma reesei*, *Bacillus licheniformis*	Baking, Food packaging	Production of white bread, Sweet potato bread, Producing biodegradable films	Hemicellulase concentration of 0.005%, 0.010%, 0.015% and 0.020% were used in dough for preparing purple sweet potato bread. pH, titrable acidity, specific volume, baking loss, color, texture and flavor of bread were examined	Dough expansion peaked with 0.015% hemicellulase, while bread pH and acidity decreased with higher levels. Lightness was highest in the control, and redness was lowest with 0.020% hemicellulase. The best overall acceptability score (5.67) was observed at 0.010% hemicellulase.	Kim et al. (2014)
L‐arabinose isomerase	*Bacillus coagulans* NL01, *B. stearothermophilus* US100	Dairy, Baking	To produce D‐tagatose – that used as a sweetener, humectant, texturizer or stabilizer	Reaction volume: 10 mL. Cell density: 4.8 g DCW L^−1^ (dry cell weight) Substrate concentration range: 20 g L^−1^ to 250 g L^−1^. Temperature Range: 40°C to 80°C	F279I Variant of l‐Arabinose Isomerase showed optimal activity at 50°C and pH 8.0 1.4‐fold higher catalytic efficiency for D‐galactose compared to the wild‐type enzyme. D‐tagatose yield, and conversion rates, making it ideal for industrial production.	Zheng et al. (2017)
Lipase	*Aspergillus oryzae*, *A. niger* , *Candida antarctica*, *A. flavus* , *C. tropicalis*	Baking, Dairy, Meat	Synthesis of flavor esters	For hydrolytic activity assay: Substrate: p‐nitrophenyl butyrate (pNPB). Solvent: 2‐propanol, pH‐8 and temperature‐25°C For enzymatic esterification Reaction Medium: Solvent (hexane, heptane, 1,4‐dioxane and cyclohexane) with butyric acid and alcohol. Substrate Concentration: 0.2–1.0 mol/L. Molar Ratios: 1:1 to 1:4 (butyric acid to alcohol). Biocatalyst: CALB‐MNP, 0.01 g (enzyme load: 80 U). Reaction Conditions: Temperature (25°C–55°C), stirring speed (50–250 rpm), volume (1 mL of solvent).	Methyl and ethyl butyrate were catalyzed by lipase B The optimum conditions for both esters were 25°C, 0.4 mol/L for ethyl butyrate, 0.5 mol/L for methyl butyrate, a 1:1 M ratio, 10 mg biocatalyst, 150 rpm, 8 h of reaction, and heptane as the solvent. The conversions rate of methyl butyrate and ethyl butyrate were more than 90%.	De Souza et al. (2017)
Rhamnosidase	*Penicillium decumbens*	Beverage	Removal of bitterness	Pretreatment: Squeezed, centrifuged, filtered with ashless filters, and homogenized with pH after being adjusted at 2.5 to 4.5 using NaOH 0.2 M. Biocatalyst: 200 mg of immobilized naringinase on Two‐Dimensional 2D zeolites. Temperature: 50°C for all experiments. Flow Rate: Adjusted to 0.25 mL/h.	High yields of sugars achieved with a contact time of just 0.83 h. Reducing sugars increased from 61% to 94%, resulting in a 52% increase in content. No enzyme deactivation observed over 300 h of operation. Turnover number of 40 g hydrolyzed naringin per gram of enzyme, indicating high enzyme efficiency. These findings confirm that the enzyme effectively reduces bitterness in grapefruit juice.	Carceller et al. (2020)
Tannase	*Klebsiella pneumonia*	Beverage	Improving the quality of fruit juices and other beverages by reducing tannin content and associated bitterness and haze.	For 10 mL orange juice, beer and tea cream solubilization clarification, optimizing enzyme concentration (0.5–7.5 U/mL), temperature (20°C–50°C), and incubation time (30–240 min)	For Orange Juice: The optimal enzyme concentration was 4.5 U/mL, incubated for 2.5 h at 35°C. Incubation for 3.5 h reduced the initial tannin content (1060 μg/mL) by 56.54%, leaving 43.46% residual tannin. For Beer: The optimal enzyme concentration for beer haze clarification was 4.5 U/mL, reducing tannin content to 71.51% after 2.5 h of incubation. Incubation at 30°C for 3.5 h reduced the initial tannin content (425 μg/mL) by 53.53%, leaving 46.47%. For Tea Cream Solubilization: At 6 U/mL enzyme concentration, tannin content in tea extract was reduced by up to 63.82% at 35°C. After 3 h of incubation, the initial tannin content (94 μg/g) was reduced by 45%	Kumar et al. (2023)
Transglutaminase	*Streptomyces mobaraensis* , *Streptoverticillium* sp.	Meat	Improves meat quality by increasing its nutritional value while reducing fat content.	1% transglutaminase and 1% papain + transglutaminase were used to formulate both beef and chicken burgers	Beef and chicken burgers treated with Transglutaminase had highest sensory scores of 6.9 for Appearance, Aroma, and Flavor, 6.3 for Texture, and an overall impression score of 7.1. Transglutaminase‐treated burgers showed superior proximate composition (fat, protein, ash) and improved physicochemical parameters (aw, pH)	Ribeiro et al. (2021)
Xylanase	*Halolactibacillus miurensis*	Baking, Brewing	Enhances the texture, shelf life, and volume of bread loaves, improving fltration	For dough fermentability, 1021.65 U/mg of Hmxyn was used in 100 g of whole‐wheat flour and fermented at 38°C and 80% RH for 0–80 min. Baked in an oven for 10 min at 180°C. The quality parameters were evaluated	Hmxyn treatment increased dough volume significantly, with 3.5 times increase at 60 min and 3.9 times increase at 80 min, 23.8% and 16.3% higher than the control, respectively. Hmxyn treatment was more effective than 60 mg Pentopon Mono BG per 1 kg flour. Specific Volume: 6 mg Hmxyn per kg flour improved bread volume; higher doses reduced it. Texture: At 6 mg, Hmxyn reduced hardness, gumminess, and chewiness by up to 35%, better than Pentopan Mono BG. Optimal Dosage: 6 mg per kg was the most effective dosage for improving bread quality.	Zhang et al. (2022)
Mannase	*Lactobacillus plantarum* (M24)	Beverage	Juice clarification	β‐Mannanase from was purified 2619.05‐fold via ammonium sulfate and DEAE‐Sephadex chromatography. Applied on apricot, orange, apple, grape, and peach juices Treatment: 10 g fruit homogenate +2 mL enzyme solution (18.6 ± 1.03 EU/mL) or 2 mL distilled water (control). Conditions: 4 h at 50°C, natural pH. Filtration: 15 min through paper filter; juice volume measured.	Mannanase significantly increased juice yield compared to the control across all tested fruits Peach juice showed the most significant improvement with a yield of 223.1% using purified mannanase and 183.15% with crude extract Enzyme treatment boosted juice yields: apple (163.11% purified, 151.5% crude), apricot (153.3% purified, 133.4% crude), grape (134.5% purified, 127.4% crude), and orange (132.5% purified, 114.6% crude) Mannanase treatment provides a cost‐effective juice production solution for large‐scale processes.	Nadaroglu et al. (2015)
Naringinase	*Aspergillus niger*	Beverage	Improve debittering of citrus juice	Substrate Preparation: Dried Citrus macroptera peel and pomace at 40°C for 48 h, stored at 5°C. Fermentation: Mixed 10 g substrate with mineral salt solution (pH 7 ± 0.2), 5% naringin, and Aspergillus niger (10^5^ spores/mL); incubated at 27°C, 120 rpm for 72 h. Enzyme Extraction: Stirred with sodium acetate buffer (pH 4.5), centrifuged (7500 rpm, 4°C) For 50 mL fresh Pomelo juice enzyme used 5 g and shaken at 220 rpm for 30 min.	Naringin Reduction: 79.76% in 120 min. Immobilized enzyme effectively debittered juice for seven cycles. Significant bioactive components were preserved with treatment times of 90–120 min. Immobilized enzyme retained 62% activity after 2 months at 4°C.	Gupta et al. (2021)
Pectinase	*Aspergillus oryzae*, *Penicillium funiculosum*	Beverage, Food, functional food	Liquefy soursop fruit pulp to yield puree, enhances the antioxidant and physicochemical properties of blueberry juice, improves the quality and yield of mulberry juice.	Pectinex Ultra SP‐L: Derived from *Aspergillus aculeatus*, for pectin hydrolysis. Enzyme Concentration: 1:5 (v/w) to mulberry mash. Incubation: 50°C, pH 4.5, for 120 min. Juice Extraction: Using a home juicer after enzyme treatment. Enzyme Inactivation: Heated to 90°C for 5 min. Pasteurization: 90°C for 10 s.	Extraction Yield (%) was peak at 120 min (87.1%), with a decline after 180 min and 240 min. Total Soluble Solids (°Bx) was 11.9 at 120 min, enhancing flavor and quality through enzyme treatment. Titratable Acidity (%) was 1.4 at 120 min, providing balanced acidity for flavor and preservation. L‐Ascorbic Acid Content (mg/100 mL) was 35.5 at 120 min, improving nutritional value with enzyme treatment. Total Phenolic Content (mg GAE/100 mL) was 160.6 at 120 min, offering high antioxidant content for health benefits. Antioxidant Capacity was 82.6 at 120 min, showing strong resistance to oxidative stress. The optimal incubation time for Pectinex Ultra SP‐L treatment is 120 min to maximize juice yield and quality.	Nguyen and Nguyen (2018)
Pentosanase	*Aspergillus* sp., *Bacillus amyloliquefaciens*	Baking	Enhancing dough and bread properties	3 types of flour (A, B, C) based on extraction rates Pentosanase levels: 0, 20, 60, and 100 ppm in each flour. Dough Mixing Process: Straight dough process with bulk fermentation (30 + 30 min) and proofing (60 min). Breads measured for weight and volume immediately after baking. Sensory and quality analysis were evaluated	At 20 ppm, pentosanase improved dough viscosity, loaf volume, and bread‐making properties. Pentosanase (20 ppm) resulted in an increase in loaf volume for this type of flour. At 100 ppm pentosanase, stability improved in flour A due to gluten coagulation, whereas flours B and C showed no significant changes Overall Sensory Quality: Positive effects with pentosanase in flour A; limited improvements in flours B and C.	Koyuncu et al. (2008)
Phospholipase	*Escherichia coli* , *Streptomyces alboflavus*	Food	Mayonnaise production	Enzyme Concentration (LEU): Experimentally tested levels were 0, 2000, 5000, 8000, and 10,000 LEU. Hydrolysis Time (minutes): Tested levels were 0, 25, 60, 95, and 120 min.	Phospholipase A2 Treatment Enhances the functional properties of egg yolk for mayonnaise production. Mayonnaise made with 6% (w/w) enzyme‐modified egg yolk (EM‐EY) showed sensory qualities comparable to mayonnaise made with 8% (w/w) fresh egg yolk (EY) while offering better stability than mayonnaise prepared with 10% (w/w) fresh EY. No significant differences were found oiliness, sourness, nutty flavor, overall acceptability, and rancid flavor between mayonnaise made with enzyme‐treated and untreated egg yolk.	Kim et al. (2009)
Protease	*Aspergillus oryzae*, *A. niger* , *Bacillus subtilis* , *A. flavus* , *Chrysosporium keratinophilum*	Beverage, Cosmetics, Detergent, Pulp and Paper, Textile, Waste management	Removing grape proteins responsible for haze formation in white wines	Pectinase Addition: 30 mg/L Flash Pasteurization: 75°C for 1 min AGP Addition: 15 mg/L Fermentation Temperature: 15°C–18°C	Aspergillopepsin I and II (AGP) effectively removed haze‐forming proteins in white wines, offering an alternative to bentonite. AGP, especially with flash pasteurization (75°C, 1 min), reduced grape proteins by about 90%, leaving non‐haze‐forming proteins unaffected. Wines treated with AGP showed no significant changes in physicochemical or sensory properties, proving it a viable alternative to bentonite fining.	Marangon et al. (2012)
Pullulanase	*Klebsiella* sp., *Bacillus* sp.	Beverage	To ensure maximum wort fermentation	Thermostable pullulanase (PersiPul1) and α‐amylase (PersiAmy2) in an 80:20 ratio Enzyme addition: 0%, 0.3%, 0.5%, 0.7%, or 1% based on total flour weight Quinoa protein isolates levels: 3%, 4%, 5%, 6%, or 7% Bread Preparation: Mix ingredients and enzyme cocktail into dough Fermentation and Baking: 1 h at 40°C and 150°C for 50 min	Thermostable pullulanase (PersiPul1) and α‐amylase (PersiAmy2) in an 80:20 ratio improved quinoa protein‐enriched bread. The enzyme mix enhanced antioxidant activity, reduced hardness and chewiness, and improved porosity, volume, browning, and sensory qualities. These results suggest the enzyme cocktail is a promising bakery bio‐additive.	Sadeghian Motahar et al. (2022)

Peptidases, or proteases, which make up around 60% of the overall enzyme market, are the dominant group in the food sector. Notable plant‐derived proteases include ficin, bromelain, papain, and keratinases (Abidi et al. 2008).

Actinidin, a cysteine protease, which was extracted from the gooseberry (kiwi fruit), significantly hydrolyzed the ester and amide linkages located on the carboxyl side of a lysine residue. In the meat industry, owing to its proteolytic properties on collagen and myofibrillar proteins, it finds widespread application as a meat tenderizer (Zhu et al. 2018). In certain dairy industries, the proteolytic activity of actinidin has also been employed to reduce antigenicity (allergenicity) and enhance functionality. Actinidin was successfully employed to reduce the antigenicity of milk proteins through the modification of protein conformation, cleavage, and masking of epitopes of β‐LG and αs1‐CN (Kaur et al. 2022). Furthermore, it can be applied in producing dairy‐based items by lowering the content of undesirable proteins (Puglisi et al. 2012).

Bromelain, a proteolytic enzyme, is isolated from water‐based extracts of pineapple stems, fruits, and foliage. Major proteases found in plant stems are known as “ananase” or “stem bromelain,” whereas proteases found in pineapple fruit juice are known as “fruit bromelain.” This enzyme finds broad application in the food sector, particularly for softening meats, baking, cheese production, seafood processing, and so on (Sharma and Vimal 2023). Since bromelain can efficiently hydrolyze meat myofibril proteins, it is primarily utilized in the food industry for meat tenderization (Arshad et al. 2014). As reported by Sarkar et al. (2017), this enzyme can also be used as an anti‐browning agent by stopping the oxidation of phenol to quinone. Additionally, it is used to improve dough relaxation, increase stability, and help avoid dough shrinkage during the baking industry (Arshad et al. 2014). In beer and wine production, this enzyme is used to enhance the protein stability of alcohol (Benucci et al. 2011).

Papain is undoubtedly one of the most extensively researched and commonly applied proteolytic enzymes in food processing. Papain is a powerful meat tenderizer because it can hydrolyze practically any protein found in muscle tissue, tendons, and ligaments. The meat‐softening effect of papain has been tested in a number of studies where the animal is given an injection of the enzyme prior to slaughter, allowing papain to be distributed uniformly throughout the meat (Bekhit et al. 2014). In baking, papain is utilized to enhance dough properties and minimize shrinkage during heat treatment (Kong et al. 2007).

### 3.3 Enzyme Production Process

**FIGURE 5:**
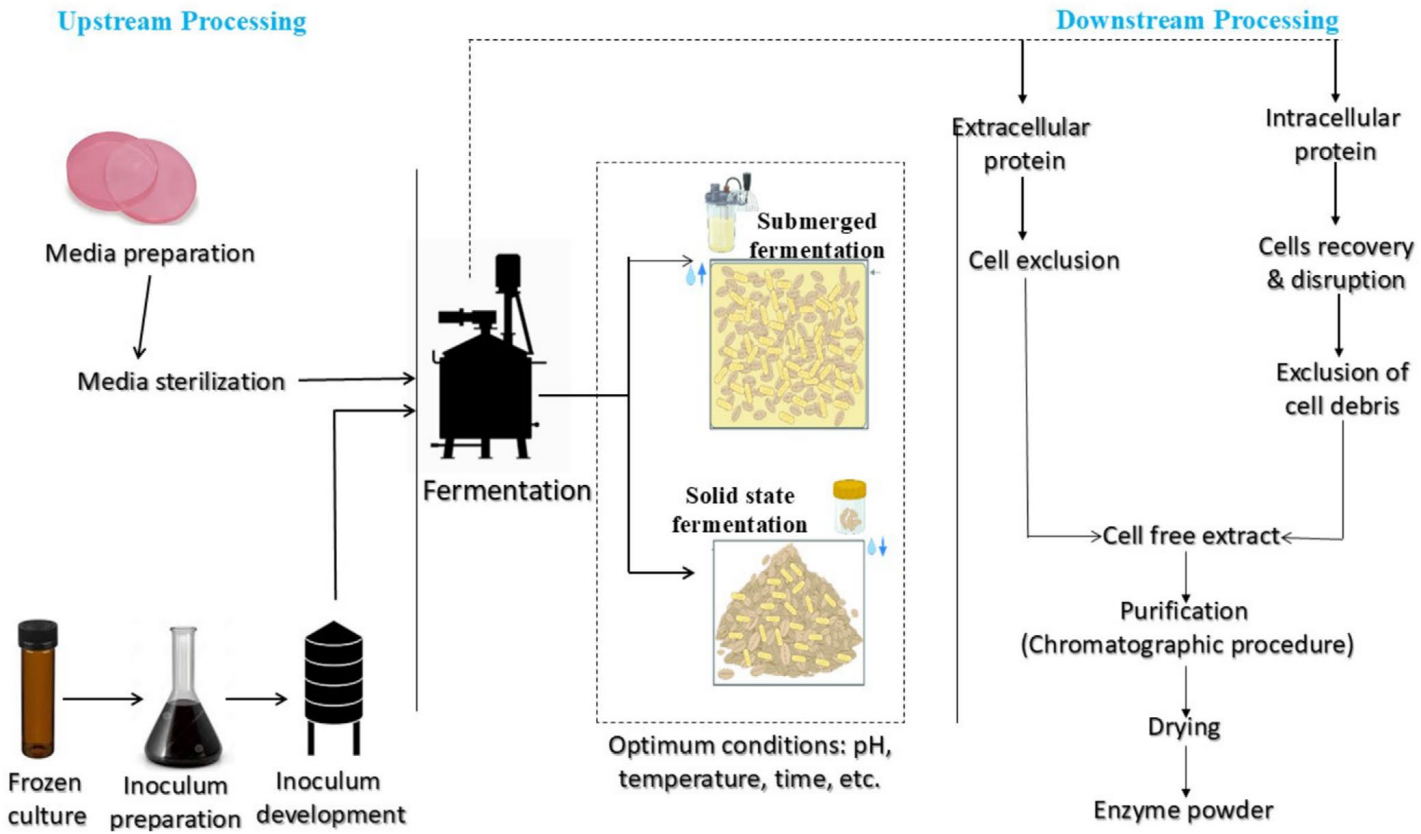
Process flow chart for commercial enzyme production.

In an industrial setting, using bacteria and fungi to produce industrially essential enzymes is preferable, since enzymes derived from microbes tend to be more stable and produce greater yields. Industrial enzyme synthesis generally follows three core stages: (i) selection of an effective microbial strain, (ii) conducting fermentation, and (iii) carrying out purification. Figure 5 illustrates the general steps involved in the production process of enzymes.

The initial and most important stage in the production of enzymes involves the careful selection of a suitable strain. Among all microorganisms, the most suitable species for this purpose are *Aspergillus*, *Bacillus*, *Streptomyces*, and *Trichoderma*, which have the capability to produce approximately 50 g/L of extracellular proteins (Patel et al. 2023). Enzyme production can occur both intracellularly and extracellularly, but the ease of purification and enzyme recovery associated with extracellular enzyme production makes it superior to intracellular enzyme production (Liu and Kokare 2023). A number of microbial enzymes, including cellulases, amylases, pectinases, laccases, lipases, and xylanases, are created outside of the cells, whereas catalase, pepsin, and peptidase are intracellular enzymes (Lu et al. 2010). In addition to strain selection, various factors such as temperature, time, type of fermentation method, pH of the media, nutrient source, and optimization of these parameters play an important role in determining the effectiveness of a fermentation process as well as achieving the desired level of enzyme production. Table 2 enlists the ideal fermentation parameters for some industrially important enzyme production.

**TABLE 2:** Ideal fermentation parameters for several industrially important enzymes.

Enzyme	Microorganism	Process conditions	Fermentation method	Optimum condition	References
Amylase	*B. amyloliquefaciens* *B. licheniformis* *Aspergillus* spp.	Incubated at 35°C–39°C Shaker incubator up to 5 days Peptone 1%–2%	SMF	Incubation time: 3 days pH = 7 Temperature: 38°C Peptone: 1.823%	Lakshmi et al. (2020)
Catalase	*Serratia marcescens*	Temperature = 30°C, Time = 2–20 h pH = 3–10	SMF	Temperature = 28°C, Time = 12 h pH = 8	Zeng et al. (2010)
Cellulase	*Trichoderma reesei*	Temperature = 30 ± 0.1°C and pH = 5.0 (Fixed) Agitation speed = 93–120 rpm Harvesting time = 31–112 h H_2_O_2_ conc. = 3.64%–10.36%	SMF	Time: 91 h Agitation speed: 120 rpm Harvesting time = 91 h H_2_O_2_ conc. = 5%	Sirohi et al. (2019)
Lipase	*Penicillium roqueforti*	Temperature = 16°C–34°C Time = 1–7 days Moisture content = 40%–88%	SMF	Temperature: 31.2°C Incubation: 1 day Moisture content: 78.8%	de Menezes et al. (2021)
Pectinase	*Streptomyces fumigatiscleroticus*	Temperature = 20°C–40°C pH = 5.0–9.0, Incubation period = 24–96 h Agitation speed = 50–250 rpm	SMF	Agitation speed = 200 rpm Temperature = 35°C pH = 6.0 Incubation period = 48 h	Govindaraji and Vuppu (2020)
Protease	*Bacillus aryabhattai*	Temperature = 30°C–40°C pH = 6.0–8.0 Inoculum volume = 1–3 v/v Maltose = 5–15 g/L Beef extract = 2.5–7.5 g/L	SMF	12.35 g/L of maltose, 5.30 g/L of beef extract, 2.5% v/v inoculum volume, pH of 7.8, and 40°C temperature	Adetunji and Olaniran (2020)
Xylanase	*Trichoderma harzianum*	pH = 4.0–6.0, Incubation period = 4–6 days Carbon source wheat bran = 0.8%–1.2%	SMF	At 70°C temperature and 160 rpm of agitation: 06 days, 5.0 pH, 1.2% wheat bran	Dhaver et al. (2022)

Once an appropriate strain has been selected and the process parameters have been optimized, the fermentation process begins for the production of enzymes on a greater scale. Two fermentation techniques can be used to manufacture microbial enzymes: SSF for fungal culture and SMF for bacterial culture. SMF is an industrial process wherein microorganisms are cultivated in a liquid nutrient medium with a high oxygen concentration in a stirred tank reactor to produce a desired product (Deckers et al. 2020). Because SMF is easier to manage and promotes superior microbial growth, it is the method of choice for large‐scale commercial enzyme synthesis. Major reaction parameters, including pH, temperature, foam, and aeration, may be controlled online using SMF without requiring the issue of heat or mass transfer (Thakur and Bhalla 2023). Therefore, it is the preferred method and is better than SSF.

In contrast to SMF, downstream processing from SSF is supposedly easier and less expensive because it is used in situations where microorganisms are cultivated in conditions with limited or no water availability. Furthermore, SSF offers several benefits, such as increased productivity, higher product concentration, reduced effluent production, and the need for basic fermentation equipment (Liu and Kokare 2023).

After fermentation, the crude enzymes need to be purified for further uses. Enzyme purification is often a complicated procedure, and to separate the residual cell debris, many techniques are typically used sequentially. Many techniques, including crystallization, electrophoresis, and chromatography, are commonly used in the purification of enzymes (Patel et al. 2023). However, it is advisable to use cost‐effective and simple approaches during the initial phases, while more advanced and expensive methods should be used in the later stages when dealing with a large volume.

## 4 Traditional Uses of Enzymes in Food Industry

Recent studies show that consumers worldwide have been increasingly opting for healthy, nutritious, and sustainable food products, not only to fulfill their basic needs but also to maintain their health. Food‐grade enzymes have a significant contribution to achieving this goal, since they are being used extensively across diverse sectors, from dairy, baking, and confectionary to functional food development within the food industry. Table 1 provides a comprehensive understanding of the usage of enzymes in different areas of the food industry.

### 4.1 Dairy Industry

The dairy industry, a crucial sector for the food industry, provides a wide range of nutritious products developed from milk, such as cheese, yogurt, butter, and milk powders, which contribute to balanced diets worldwide. To enhance the quality of dairy products, including improved flavor, texture, color, and nutritional properties, and to standardize the production process for consistent results despite variations in milk composition, enzymes are used in the food sector. Additionally, enzymes can facilitate the utilization of alternative milk sources, such as plant‐based or non‐traditional animal milks, thereby reducing the environmental impact of dairy production. Additionally, 70% of people on the planet are lactose intolerant and suffer from symptoms including bloating, diarrhea, nausea, and stomach discomfort after eating lactose‐containing foods (Xavier et al. 2018). Using the lactase enzyme, lactose may be hydrolyzed into glucose and galactose to provide lactose‐free dairy products (Dekker et al. 2019). Enzymatic treatment is also used in the production of several additional lactose‐free dairy products in addition to lactose‐free milk. One such product made with a similar method is flavored milk (Arruda et al. 2013).

Widely used in sectors like dairy, lipase is an enzyme with strong catalytic activity that is becoming more and more popular, especially for taste creation. Lipase has been used in the dairy sector for cheese production, taste improvement, and lipolysis of milk and butterfat. When making cheese that has been treated with enzymes, lipases are used to give the cheese a concentrated taste.

Since ancient times, rennet—a mixture of the proteolytic enzymes chymosin and pepsin—has been utilized in the manufacturing of cheese. Different forms of rennet, such as plant, fungus, and animal rennet, are used in the production of cheese and are categorized according to their source. Because of its higher heat stability and increased stability between 4.0 and 6.0 pH without losing activity, rennet derived from *Mucromiehei* produces cheddar cheese of exceptional quality, even with a high ripening process (Agarwal et al. 2022).

### 4.2 Food and Beverage Industries

The beverage industry is becoming one of the largest food‐processing industries, as there has been growing consumer demand worldwide in recent times. To meet the growing global demand, the highly fragmented beverage industry produces a wide variety of products, broadly classified into alcoholic drinks (wine, beer, and whiskey), nonalcoholic beverages (soft drinks and fruit juices), coffee, and tea.

The beverage industry faces several challenges while producing beer, wine, and clear fruit juices, such as fouling issues during filtration caused by polysaccharides and haze development due to protein–polyphenol interactions, resulting in the generation of polymer‐like aggregates (Uzuner and Cekmecelioglu 2019). Over time, commercially, fruit juices are clarified using fining chemicals such as bentonite and gelatin (Bhattacharjee et al. 2017). However, in recent times, enzymes are used in place of chemicals to enhance sedimentation and juice clarification while also improving extraction performance by boosting both yield and overall process effectiveness (Cosme et al. 2023). Moreover, with advancements in biotechnology, commercially produced enzymes are extensively applied at different stages of beverage manufacturing to enhance product quality while simultaneously minimizing energy use, greenhouse gas emissions, and ecological impact (Claus and Mojsov 2018; Mazrou et al. 2020). Amylases, cellulases, and pectinases are the most widely used enzymes in the beverage industry across various food and beverage enzyme categories (Uzuner and Cekmecelioglu 2019).

For the production of beer, enzymes are essential for converting starch into fermentable sugars, controlling viscosity, aiding in fermentation, enhancing flavor and color, producing low‐calorie beers, and reducing colloidal haze during chill‐proofing (Van Donkelaar et al. 2016; Uzuner and Cekmecelioglu 2019; Carvalho et al. 2023). Amylase and glucoamylase are key enzymes used during malting and fermentation to break down starch into fermentable sugars, enhancing yeast fermentation and enhancing the texture, taste, and appearance of the end product (Singh, Kundu, et al. 2019). Additionally, glucoamylase, derived explicitly from *Aspergillus* species, is directly added during fermentation to produce sweet beers without needing added sweeteners by digesting dextrins and eliminating non‐fermentable carbohydrates (James and Lee 1997). In the chill‐proofing stage, proteolytic enzymes such as papain or protease are employed to reduce colloidal haze caused by polypeptide and polyphenolic complexes, which results in a clearer, more stable beer (Wang and Ye 2021). In the wine industry, enzymes are extensively utilized in different production stages, from clarification and color extraction to maceration and protein stabilization. Enzymes such as pectinases, hemicellulases, and cellulases are particularly important for enhancing the yield of must during pressing, improving overall processing efficiency, and improving product quality (de Souza and Kawaguti 2021). While producing white wines, pectic compounds released during grape pressing develop into colloidal particles that can affect aroma and flavor. Enzymatic hydrolysis using pectinases is essential to break down these particles, improving clarity, reducing filtration time, and increasing must volume (Espejo 2021).

### 4.3 Food Analysis

**FIGURE 6:**
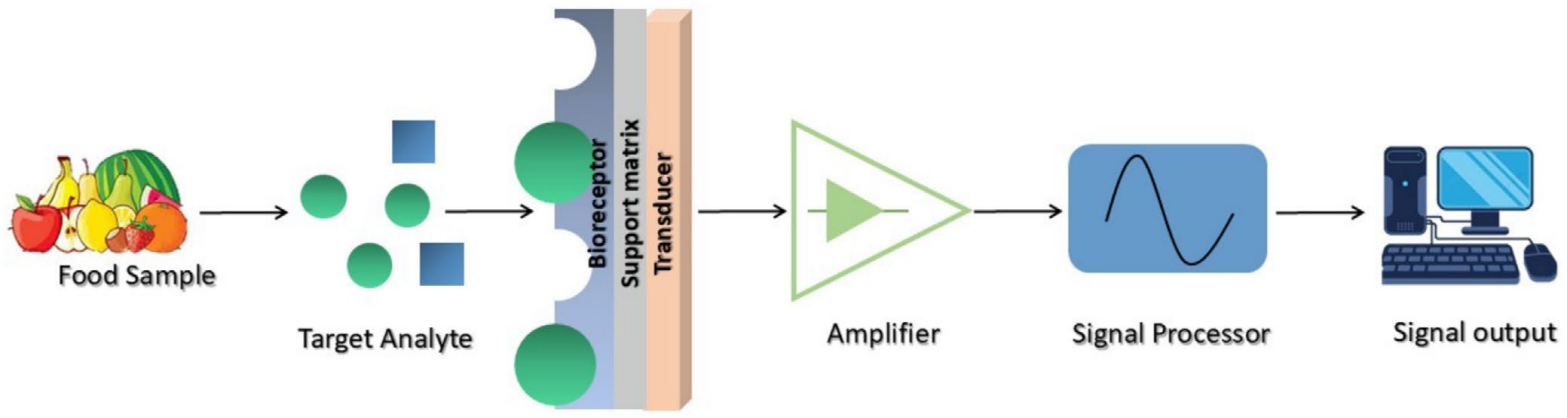
Schematic representation of an enzyme‐based biosensor.

Enzymes are widely used in food analysis, where they are employed as a cost‐effective, rapid, and reliable analytical tool to identify allergens and foodborne pathogens. A biosensor, an enzyme‐based analytical tool, plays a vital role in evaluating food safety and quality. Basically, biosensors utilize biological components, specifically a bioreceptor and a transducer, to turn the response of the bioreceptor into an analytical signal. Figure 6 describes the working principle of a biosensor.

Food products are inherently perishable; unscrupulous traders often intentionally add adulterants like urea, melamine, and other preservatives to compromise food quality for financial benefit. To detect these adulterants in food samples, enzyme‐based biosensors have been developed. For example, Ramesh et al. (2015) developed a biosensor based on immobilized *Arthrobacter creatinolyticus* urease and showed its feasibility for detecting urea in milk samples. Moreover, to simultaneously determine picomolar concentrations of melamine and urea in cow milk samples, Ezhilan et al. (2017) introduced an acetylcholinesterase cyclic voltammetric biosensor.

Nowadays, foodborne illnesses are arising at an alarming rate, which can arise from the production of various toxins (aflatoxins, mycotoxins) by pathogenic microorganisms that attack food during food preparation, packaging, and storage. A horseradish peroxidase (HRP)‐based biosensor has been developed for the determination of the mycotoxin ochratoxin A (OTA) by using the enzyme immobilization process (Alonso‐Lomillo et al. 2011).

Histamine, a biogenic amine whose increased concentration is considered an indicator of decreased freshness levels, is most commonly associated with intoxication in individuals consuming fish products. Researchers employed the histamine oxidase enzyme derived from *Arthrobacter crystallopoietes* to develop a biosensor capable of detecting histamine concentrations in meat and fish products (Rosini et al. 2014).

The development of biosensors requires much research to gain the necessary characteristics, such as a low detection limit, a quick response time, and long‐term stability, that every biosensor must have to be used commercially. In the future, it is expected that biosensors will quickly replace traditional techniques for evaluating food quality because of their increased portability, simplicity, and improved sensitivity and reproducibility.

## 5 Sustainable Applications of Enzymes in the Food Industry

### 5.1 Food Industrial Wastes Valorization

In recent years, the food‐processing sector has emerged as one of the most rapidly expanding global industries, and substantial quantities of waste are generated throughout food manufacturing operations. The fruit and vegetable sector produces higher levels of waste than many other branches of food processing, which include peels, seeds, stones, pods, skins, and pomace (Ajila et al. 2010). Managing and disposing of this waste often adds to the total production expenses. As a result, this waste has become a critical environmental concern, contributing to air and water pollution as well as greenhouse gas emissions due to its inefficient usage and absence of eco‐friendly recycling practices (Sarker et al. 2024).

Waste generated by the food sector is a valuable reservoir of key biologically active compounds, minerals, and nutrients comprising components like carbohydrates, fiber, proteins, lignin, cellulose, hemicellulose, pectin, fats, and antioxidant molecules (Ng et al. 2020; Hasan et al. 2024). So, waste valorization can be an effective process for extracting nutrients and producing valuable products, such as fuels and materials, along with additional high‐value products, supporting both ecological and financial sustainability. In this case, enzymes serve an important function in circular economic models by converting food waste into beneficial secondary products, offering a novel and eco‐friendly approach to addressing worldwide waste disposal issues (Nargotra et al. 2023, 2024).

Enzymatic processes have significant potential in lignocellulosic biorefineries by producing advanced biofuels that offer an eco‐friendly substitute for traditional petroleum‐based fuels (Singh et al. 2022). Enzymatic hydrolysis and fermentation of structural carbohydrates like cellulose and hemicellulose effectively break them down into sugars suitable for fermentation, which are subsequently transformed into ethanol and biodiesel via microbial bioconversion (Singh et al. 2022; Sharma et al. 2023, 2024). Several commercial enzymes, including cellulases and amylases, facilitate the breakdown of starches and complex polysaccharides to liberate sugars usable in fermentation (Singh et al. 2024). Enzymatic valorization of food waste can occur through processes like oxidation, hydrolysis, esterification, and anaerobic digestion (AD). AD converts food waste into methane and biohythane, while lipids in food waste are transformed into biodiesel using bioconversion technologies (Lam et al. 2015; Pedro et al. 2024). For example, immobilized *Bacillus subtilis* lipase on magnetic nanomaterials generates biodiesel from food‐based residues such as leftover olive oil (Maroju et al. 2023). Anaerobic digestion is a highly efficient, environmentally friendly method for treating food waste, which offers the dual benefits of generating biomethane and reducing solid waste. However, the rate‐limiting step of hydrolysis restricts biomethane yield (Chen et al. 2024). Enzymatic pretreatment using proteases and amylases accelerates the breakdown of complex food waste components, improving biomethane production (Zou et al. 2020).

### 5.2 Clean in Place

Proper cleaning is essential for maintaining quality control in food processing, as neglecting to clean equipment adequately can raise processing costs by reducing the heat transfer efficiency and posing a microbial contamination risk in the industry (Dallagi et al. 2023). One of the most widely used systems for cleaning and sanitizing equipment, processing lines, and storage units without the need for disassembly is Cleaning‐in‐Place (CIP) (Moerman et al. 2014). CIP systems are commonly employed in the dairy industry and various other food sectors, including breweries and the production of edible oils and fats, where they are used to clean pipe‐based systems by eliminating deposits and fouling (Guerrero‐Navarro et al. 2019). However, CIP systems have high demands for water, chemicals, and energy, which lead to the production of large volumes of wastewater (Moerman et al. 2014). This wastewater not only adds to the operational costs for the industry but also places an environmental strain on the surrounding community (Boyce et al. 2010).

Currently, novel strategies such as enzyme‐based CIP approaches have emerged as promising alternatives to traditional chemical‐based CIP cleaning agents in the dairy industry. These methods employ enzymes to degrade organic residues on processing surfaces, minimizing the reliance on harsh chemicals and enhancing sustainability (Pant et al. 2023).

Guerrero‐Navarro et al. (2019), using proteases and amylases in a lab‐scale milk fouling model, demonstrated that enzyme‐based CIP was more effective than traditional cleaning products in reducing fouling, cleaning time, energy consumption, and wastewater generation (Guerrero‐Navarro et al. 2019). Tests on various commercial enzymes revealed that Esperase, Properase, and Savinase demonstrated the best performance, with Properase and Savinase identified as potential candidates for dairy industry applications (Selamoglu 2020). Immobilization techniques also improved enzyme activity and reusability, as shown by Paul et al. (2014), suggesting the potential for industrial‐scale application (Paul et al. 2014).

Biofilm formation, another significant issue in dairy processing, arises when bacteria adhere to surfaces such as process tanks, lines, and heat exchangers, leading to contamination and fouling (Gonçalves et al. 2020). Pathogenic bacteria such as *Listeria monocytogenes* , *Escherichia coli* , and *Salmonella* spp. frequently form biofilms in dairy environments, making them resistant to conventional disinfection methods (Gonçalves et al. 2020). To overcome the limitations of chemical cleaning, enzyme‐based approaches have emerged as potential alternatives. Enzyme‐based treatments, such as those combining oxidoreductases with polysaccharide‐hydrolyzing enzymes, have been effective against biofilms of pathogenic organisms on surfaces like polypropylene and steel (Boyce et al. 2010). The use of enzymes in biofilm removal offers several advantages, including specificity, fast reaction rates, and the ability to function under moderate conditions. However, more research is needed, particularly in mixed‐species biofilms and industrial‐scale applications (Pant et al. 2023). Classes of enzymes with potential for biofilm removal include proteases, cellulases, lipases, amylases, and DNases. Combining enzymatic treatments with traditional disinfection methods could offer a more effective strategy for managing biofilms in dairy processing environments (Pant et al. 2023).

### 5.3 Extraction of Bioactive Compounds

Bioactive compounds are phytochemicals present in foods that have the potential to influence metabolic processes and contribute to improved health. These compounds, found in fruits, vegetables, and whole grains, include a wide variety of compounds, notably carotenoids, polyphenols, carotenoids, tocopherols, phytosterols, and organosulfur compounds (Łubek‐Nguyen et al. 2022). Most bioactive compounds show antioxidant, cancer‐preventive, anti‐inflammatory, and antimicrobial effects, and epidemiological studies suggest that many also offer protective effects against cardiovascular diseases (Hamzalıoğlu and Gökmen 2024).

Bioactive compounds are commonly extracted using conventional solid–liquid or liquid–liquid extraction methods, such as Soxhlet extraction, a method employed for many years. However, these traditional techniques often take a long time, demand significant solvent volumes, and result in higher operating costs as well as environmental issues (Uzuner and Cekmecelioglu 2019). As a result, the efficient recovery of bioactive compounds from natural origins is highly valued across industries, with an increasing emphasis on the use of economically and environmentally friendly technologies.

Bioactive compounds in natural sources, such as plants and fungi, are often encapsulated within complex cell wall structures that serve as barriers. Cell walls in plants primarily consist of polysaccharides such as pectin, cellulose, and hemicellulose, providing mechanical strength and protection, while secondary cell walls offer additional reinforcement (Łubek‐Nguyen et al. 2022). Effective hydrolysis of these cell wall components can improve the release of bioactive ingredients through enzyme‐assisted methods (Rani et al. 2021). Enzyme‐assisted extraction (EAE) presents an eco‐friendly and cost‐effective alternative to traditional solvent‐based methods for obtaining nutraceuticals and bioactive compounds from natural sources. EAE reduces extraction time, minimizes solvent use, and enhances yield and product quality (Al‐Hemaid and Chandrasekaran 2015). Disca et al. (2024) showed that enzymatic treatments of cocoa bean shells (CBSs) significantly increased the soluble dietary fiber (SDF) to insoluble dietary fiber (IDF) ratio without affecting antioxidant activity or bioactive compounds, highlighting their potential for valorization in the food and nutraceutical industries (Disca et al. 2024).

Still, more research should focus on advancing enzymatic processes to improve bioactive compound yields and evaluate their impact on the biological availability and effectiveness of nutraceuticals, as enzymes remain underutilized in nutraceutical development.

### 5.4 Functional Food Development

Functional foods are foods or dietary ingredients that offer health advantages beyond just supplying the body with the nutrients it needs. Functional foods can be broadly categorized as foods that have been fortified, whole, enriched, or enhanced and that, when regularly consumed in sufficient amounts, offer physiological health benefits beyond the supply of essential nutrients (e.g., vitamins and minerals) (Alemzadeh 2020; Miah, Chy, Ahmed, et al. 2025). At present, consumers are becoming more aware these days, keeping an eye on diets that use new and easily accessible foods that make use of cutting‐edge technologies (Fuentes‐Zaragoza et al. 2010). The use of functional foods has increasingly been applied to enrich beneficial gut microbiota, thus promoting health, relieving symptoms associated with gut‐related diseases and side effects of conventional treatments, and improving overall well‐being (Dixit et al. 2023). Enzymes, including lipase, protease, chlorophyllase, tannase, L‐asparaginase, and beta‐glucanase, play a crucial role in the manufacturing of nutraceuticals and functional foods. Enzymes used in functional food also contribute to sustainability by reducing the need for artificial additives and promoting the use of bioactive compounds, which makes the food industry more eco‐friendly and health‐conscious.

A group of biological compounds known as “bioactive peptides” is made up of molecules that are often encrypted in the structure of parent proteins and become active upon release (Akbarian et al. 2022). The proteolytic action of enzymes such as trypsin, pepsin, and chymotrypsin results in the alteration of the molecular conformation of native proteins, thereby releasing bioactive peptides (Alemzadeh 2020). These functional ingredients have a wide range of health benefits, including antimicrobial and antioxidant activities, blood‐lipid‐lowering effects, anti‐obesity properties, antiaging, and antidiabetic effects (Akbarian et al. 2022). According to (Phelan et al. 2009), the health benefits of biologically active milk bioactive peptides released from casein hydrolysates include lipid oxidation inhibition, radical scavenging, antiproliferation, antimicrobial activity, antimutagenicity, and immunosuppression. In cheesemaking, the acceleration of the ripening process helps to increase the amount of bioactive peptides. A study shows that encapsulation of protease enzymes in liposomes can accelerate cheese ripening, which in turn increases bioactive peptides (Zoghi et al. 2018; Mandal et al. 2025). Beyond cheese ripening, enzyme encapsulation has become a valuable strategy in functional food development, enhancing enzyme stability during processing, protecting against gastrointestinal conditions, and enabling controlled release (Weng, Li, et al. 2024). For instance, encapsulation of lycopene using techniques such as spray‐drying, molecular inclusion with β‐cyclodextrin, microencapsulation with proteins and gums, and supercritical CO₂ extraction helps protect it from degradation and oxidation, thereby improving its stability and efficacy (Bakhshizadeh et al. 2025).

Another useful enzyme for the food industry is lipase, which can be used to produce fatty acid derivatives such as antioxidant‐rich fatty acid esters, flavoring esters, and structured lipids (Ferreira et al. 2013). These enzymes are mainly used for dairy processing, primarily to promote cheese maturation and break down milk lipids to enhance flavor in cheese products (Afarin et al. 2018). Dietary fat quality plays a crucial role in preventing and managing early‐onset obesity, high blood pressure, diabetes, and cancer. Hence, conjugated fatty acids are well‐studied for their ability to reduce the likelihood of chronic illnesses (Niezgoda and Gliszczyńska 2019). According to Guo et al. (2024), lipases could be applied in the large‐scale synthesis of conjugated linoleic acid.

Polyphenolic compounds are a diverse group of plant‐derived secondary metabolites known for their beneficial health attributes, including their crucial role in reducing degenerative diseases (Albuquerque et al. 2021). However, the development of complexes with macromolecules like proteins, carbohydrates, and minerals affects the bioavailability and bio‐efficacy of polyphenolic compounds, thereby limiting their uptake within the intestinal tract (Kuddus 2019a). Enzymatic hydrolysis, mainly catalyzed by tannase, an extracellular enzyme, can break down complex polyphenols into simpler substances like gallic acid and related tannin oligomers (Aharwar and Parihar 2019). These simpler compounds have a higher absorption rate in the small intestine, which provides several beneficial properties, such as antioxidant effects, inflammation reduction, and cancer prevention potential.

Consequently, enzymes contribute greatly to food biotechnology, especially in developing and marketing functional food products. However, it is important for researchers to keep in mind that clinical trials, as well as in vitro and in vivo investigations, are necessary measures that must be taken into consideration in order to guarantee that there will be no adverse health effects.

## 6 Current Trends and Innovations for Enzymes in the Food Industry

**FIGURE 7:**
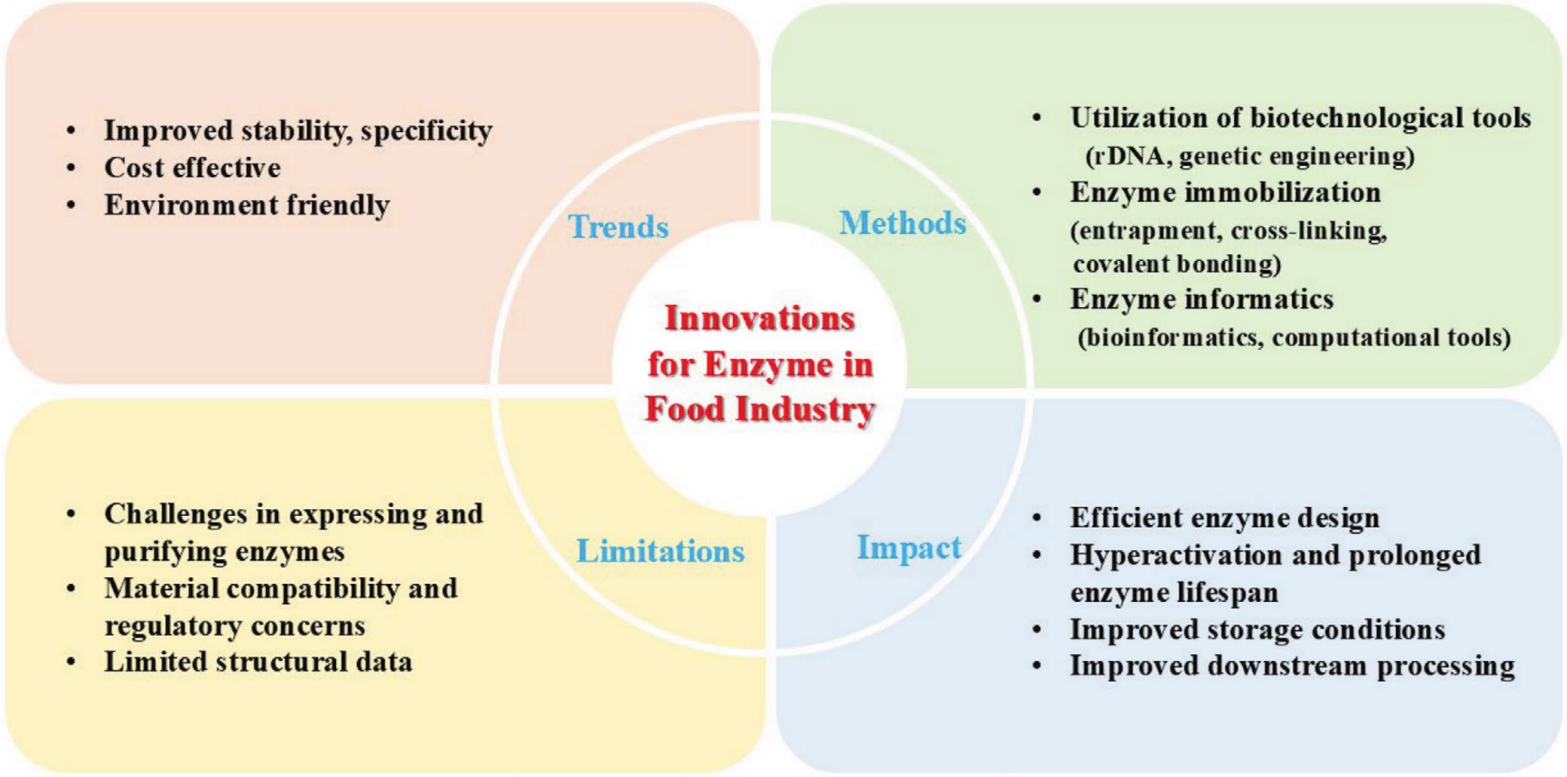
Trends, methods, limitations, and impacts of enzyme innovations in the food industry.

Recently, innovative enzyme technologies have created opportunities for applying enzymes to make food processing more cost‐effective and eco‐friendly. Today, the implementation of gene technology for the production of novel industrial enzymes, as well as the optimization of existing proteins, offers several advantages in food industry applications, such as enhancing product quality and minimizing reliance on primary resources, replacing artificial additives, and also helping avoid harmful food by‐products (Boukid et al. 2023). Moreover, genetic engineering improves extraction and purification of enzymes and mass production with decreased costs and minimized energy input from microbial or plant sources, making them more suitable for food manufacturing and processing (Fasim et al. 2021). Different biotechnological tools, such as screening, rDNA technology, and protein engineering, using single or integrated approaches, have been developed to enhance desirable traits in enzymes, such as stability at high temperatures, the ability to work in a wide range of pH levels and pressures, fast reaction rates, and the ability to function in the presence of typical inhibitory molecules (Rastogi and Bhatia 2019). Enzyme immobilization allows for high activity and productivity in bioreactors, simplifies downstream processes through easy recovery and reuse of biocatalysts, enables continuous or automated operation, and makes enzyme use more economical with minimal or no loss of activity (Fernandes 2010). Figure 7 provides a comprehensive overview of the trends, methods, limitations, and impacts associated with enzyme innovations in the food industry.

### 6.1 Biotechnological Tools

Utilization of genetic engineering or recombinant DNA technology can tailor both novel and known enzymes to achieve desired specificity and sensitivity while also reducing production costs (Srivastava 2019). Microorganisms represent a highly promising source with significant potential for obtaining food enzymes. The use of recombinant DNA technology enhances microbial sources, making them more cost‐effective, adaptable, and suitable for large‐scale industrial applications (Srivastava 2019). Advancements in microbial strain development have enabled the enhancement of enzyme yields by eliminating native genes responsible for extracellular protease production. These engineered strains not only simplify the enzyme extraction and purification process but can also be cultivated using cost‐effective raw materials, thereby making enzyme production more sustainable (Rastogi and Bhatia 2019). For instance, recombinant *Bacillus subtilis* α‐amylase, expressed with a His‐tag for enhanced purification, exhibited improved biochemical properties and enzyme activity compared to its native form, and its application in bread‐making significantly enhanced dough texture and bread quality (Salem et al. 2024).

Screening methods focus on isolating enzymes from extremophiles to ensure that the enzymes can withstand harsh conditions in industrial applications while maintaining stability, high activity, specificity, and efficient turnover rates (Boehmwald et al. 2016). Thermophiles, particularly those from high‐temperature environments like hydrothermal vents, are frequently used in the food industry for starch hydrolysis, utilizing enzymes such as amylopullulanases, alpha amylase, fructosyltransferases, glucose (xylose) isomerases, glucoamylases, glucosidases, levansucrases, inulinases, pullulanases, xylanases, and β‐galactosidases (Fernandes 2010; Hossain and Alam 2022; Tafolla‐Arellano et al. 2024). For example, a study investigated thermophilic bacteria from Ethiopian hot springs, isolating and screening 252 bacterial strains for their production of thermostable hydrolytic enzymes like amylase, cellulase, protease, and lipase. The findings highlight the potential of these hot springs as a source of industrially significant thermostable enzymes, with *Bacillus licheniformis* being the dominant species (Guta et al. 2024). Moreover, Rathakrishnan and Gopalan (2022) isolated and characterized moderately halophilic bacteria from Indian salterns, identifying various genera such as *Bacillus*, *Staphylococcus*, and *Pseudomonas*, which produce hydrolases like amylase, lipase, protease, and cellulase. Notably, *Bacillus paramycoides* was discovered to produce lipase, and these salt‐tolerant extracellular enzymes have significant potential for industrial applications (Rathakrishnan and Gopalan 2022).

Protein engineering, another key biotechnological tool, is essential for improving the catalytic activity of enzymes, including substrate compatibility and specificity under conditions suitable for industrial use (Rastogi and Bhatia 2019). At present, protein engineering primarily utilizes two established methods: directed evolution and rational design, which are primarily employed to develop enzyme variants with enhanced performance, expanded substrate range, and greater substrate specificity (Hossack et al. 2023). Rational design depends on existing structural and functional knowledge of proteins to implement targeted changes via site‐specific mutagenesis (Oroz‐Guinea et al. 2018). Conversely, directed evolution operates without needing prior data and introduces random mutations using chemical agents or error‐prone PCR techniques (Nirantar 2021). Nevertheless, both strategies encounter major obstacles due to their high cost, labor intensity, and experimental complexity of experimentally screening the vast protein sequence space. To address these challenges, machine learning (ML) has been applied in protein engineering, which results in the development of numerous protein variants with improved catalytic activity and selectivity (Alfonzo et al. 2022).

Machine learning techniques help predict how protein variants will perform by using limited experimental data, which provides a more efficient way to guide protein engineering efforts (Alfonzo et al. 2022). This data‐driven approach narrows down the most valuable parts of the protein sequence to explore, reducing unnecessary testing. Additionally, machine learning can detect important patterns, such as complex interactions between mutations, that might be overlooked otherwise (Ao et al. 2024).

### 6.2 Enzyme Immobilization

Immobilization is a process where enzymes are fixed to solid supports to create a heterogeneous system that mimics their natural attachment to cellular structures like the cytoskeleton, membrane, and organelles in living cells (Ji et al. 2024). An immobilized enzyme can be a free enzyme, an organelle, a cell, or a combination of these and can be repeatedly and continuously used in various food industrial processes, as well as in bioaffinity chromatography, biosensors, and various diagnostic applications (Mandake et al. 2020).

There are several techniques for enzyme immobilization, such as binding, entrapment, and cross‐linking (Adhikari 2019). Binding to support involves physical, ionic, or covalent bonding, where enzymes attach to carriers like agarose gels through weak forces such as Van der Waals or form strong covalent bonds with functional groups on the enzyme's surface (Sirisha et al. 2016). Entrapment encases the enzyme within a polymeric network, allowing substrates and products to diffuse while keeping the enzyme confined. However, this method may affect reaction kinetics due to mass transfer limitations (Kaushal et al. 2022). In cross‐linking, enzymes are chemically bonded to the support, creating a stable three‐dimensional structure, with agents like glutaraldehyde forming links through amino groups, enhancing enzyme stability and reusability in various industrial applications (Kaushal et al. 2022).

Enzyme immobilization offers several advantages, including hyperactivation and increased stability through multipoint or multi‐subunit attachment (Bilal and Iqbal 2020). It can also enhance the enzyme's activity, specificity, and selectivity while improving both thermal and operational stability and storage (Bilal et al. 2018). These properties make immobilized enzymes well‐suited for challenging and fluctuating industrial environments. Additionally, immobilization streamlines downstream processes by facilitating easier separation and purification of products from the biocatalyst (Bilal and Iqbal 2020). Immobilized enzymes can be recovered and reused from complex reaction mixtures, which enhances productivity and minimizes substrate inhibition.

Recent advancements have shown the effectiveness of encapsulating enzymes within biocompatible matrices to enhance thermal stability, control release, and scale up for industrial applications. For instance, phytase encapsulated in alginate using spray‐drying significantly improved both thermal (up to 80%) and storage stability (6‐month retention: 93%), indicating promise for large‐scale food and feed applications (Weng et al. 2023). Similarly, encapsulating enzymes in metal–organic frameworks (MOFs), particularly MIL‐88A(Fe), has enabled improved thermal endurance (up to 95°C) and controlled release through defect engineering, offering potential for food‐grade applications due to its non‐toxicity (Weng, Xu, et al. 2024; Weng, Yan, et al. 2024). These encapsulation strategies are especially promising for overcoming challenges of enzyme fragility and scalability in food‐processing environments.

Immobilizing SM2 xylanase via cross‐linking enhances its thermostability, shifts its optimal pH to 5, improves reuse efficiency, and eliminates the need for refrigerated storage, while also effectively producing xylooligosaccharides with potential prebiotic applications in the food industry (Fernandes et al. 2024). Another one is that immobilizing L‐asparaginase from *Pseudomonas aeruginosa* HR03 on chitosan nanoparticles significantly improves enzyme stability, extends its optimal operating temperature and pH, and enhances long‐term durability, making it a promising candidate for applications in the food and pharmaceutical industries (Baluchi and Homaei 2024).

So, the progress in enzyme immobilization techniques significantly contributes to achieving sustainability by optimizing resource use and minimizing waste, thereby broadening their application scope across diverse industrial sectors (Maghraby et al. 2023).

### 6.3 Enzyme Informatics

In today's world, enzyme engineering increasingly relies on bioinformatics and computational tools, which provide valuable insights into enzyme structure and functionality. Enzymes, as central components of metabolic reactions, are essential for the survival of all organisms. The rapid advancement of technologies such as next‐generation sequencing (NGS), X‐ray crystallography, mass spectrometry, and nuclear magnetic resonance (NMR) has generated huge amounts of structural and sequencing data. Bioinformatics tools enable efficient analysis of this data to allow researchers to interpret enzyme functionality and structure–activity relationships with unprecedented speed and accuracy (Rastogi and Bhatia 2019; Ogunjobi et al. 2024).

Several databases, including NCBI RefSeq, UniProt, and PDB, provide comprehensive resources for enzyme information, such as sequence and 3D structural data (Das and Ghosh 2024). Additionally, specialized databases such as BRENDA, KEGG, and ExplorEnz offer detailed enzyme‐specific information, including kinetics, biological sources, and roles in metabolic pathways (Dutt and Meghwanshi 2024). These interconnected databases streamline the retrieval and analysis of enzyme data, facilitating the understanding of enzyme function and catalysis in biological systems.

In enzyme engineering, two primary approaches are employed: de novo enzyme design and enzyme redesign. The de novo approach focuses on the creation of novel enzymes with specific catalytic properties, while enzyme redesign aims to improve the activity, specificity, and stability of naturally occurring enzymes (Braun et al. 2024; Zheng et al. 2024). Advanced software tools, such as ROSETTA, ORBIT, WISDOM, and EVODESIGN, have been developed to aid in both approaches, enabling the rational design of enzymes for non‐biological reactions or improved functionality (Rastogi and Bhatia 2019; Kaushik et al. 2023). Although designed enzymes often do not yet match the efficiency of natural enzymes, further optimization is possible through directed evolution techniques, which refine catalytic performance.

These tools, combined with bioinformatics databases and computational approaches, are transforming enzyme engineering, facilitating the development of tailored enzymes for diverse industrial applications.

## 7 Novel Food Enzymes

The distinctive features of novel enzymes are thermostability, pH adaptability, low temperature activity, and tolerance to high pressure, metal ions, salinity, inhibitors, and solvents, which make them invaluable assets in food bioprocessing (Zhang et al. 2018). Table 3 highlights the distinctive characteristics, working mechanisms, and advantages of novel enzymes over traditional enzymes.

**TABLE 3:** Characteristics, mechanisms, and benefits of novel enzymes compared to traditional enzymes.

Category of novel enzymes	Mechanism of action	Enzymes	Source microorganism	Benefits over traditional enzymes	References
Thermophilic	Increased hydrophobic interactions, ionic bonds, and compact folding to maintain activity at high temperatures.	Glucoamylases	*Bacillus licheniformis* , *Thermoanaerobacter tengcongensis* , *Picrophilus torridus*	Potential for using starch industry where high heat requires	Zheng et al. (2010), Ghani et al. (2013), Ashaolu et al. (2025)
		Proteases	*Halobacterium* sp. strain HP25, *Thermoascus aurantiacus*, *Anoxybacillus caldiproteolyticus*	Highly stable against temp., pH, salt, and organic solvents	Han et al. (2023), de Melo et al. (2024)
		Laccases	*Bacillus* sp.	Alternative to physical–chemical adsorbents in wine industry	Edoamodu and Nwodo (2024)
Psychrophilic	Enhanced flexibility of active sites and reduced structural rigidity to catalyze reactions at low temperatures.	Amylases	*Alteromonashaloplanktis*, *Aeromonasveronii* NS07, *Saccharophagus degradans*	Significantly halotolerant	Srimathi et al. (2007), Samie et al. (2012), Wayllace et al. (2021)
		β‐Galactosidases	*T. frigidphilosprofundus*, *Cryobacterium* sp. LW097, *Alkalilactibacillus* sp.	Improve process stability by cutting costs	Ghosh et al. (2025)
		Pectinases	Aspergillus sp., Bacillus sp., *Aureobasidium pullulans*	Help to cook meals at low temp.	Hidayah et al. (2020), Oskay (2022)
Acidophilic	Proton exclusion mechanisms, stable ionic networks, and membrane impermeability to function in low pH.	α‐Amylases	*Bacillus* sp. YX‐1, *B. amyloliquefaciens*	Eliminates the need for pH adjustment and calcium ions	Ngalimat et al. (2021)
		Glucoamylases	*Picrophilusoshimae*, *Thermoplasma acidophilum* , and *P. torridus*	Efficient starch hydrolysis, and suitability for producing low‐calorie product	Abd‐Elhalem et al. (2015)
		Proteases	*Bacillus* strain NTAP‐1	Use in collagen hydrolysis without requiring pH adjustments.	Sorapukdee et al. (2020)
		Xylanases	*A. capsulatum* , *S. solfataricus*	Efficient in the saccharification of xylan and cellulose into fermentable sugars.	Curci et al. (2021)
Alkaliphilic	Increased arginine and histidine residues with decreased glutamate content for stability in high pH environments.	α‐Amylases	*Bacillus* strain	Can hydrolyze starch into maltotriose, maltose, and glucose	Roy et al. (2013)
		Cyclomaltodextrin Glucanotransferases	*Bacillus stearothermophilus*	Cyclodextrin production at high pH and temp. with reduced waste and costs.	Shibuya et al. (2004)
		Lipases	*Bacillus* sp.	Acted well on natural fats, oils, and triglycerides with fatty acids	Vaishnavi et al. (2023)
		Pectinases	*Bacillus* sp. strain GIR 277	Have significant degumming capacity	Vaishnavi et al. (2023)
Halophilic	Maintenance of osmotic balance through ionic shields and high surface charge to function in saline conditions.	Xylanases	*Glaciecola mesophila* , *Chromohalobacter* sp., *Nesterenkonia* sp	Use in bioethanol production as cellulosic materials	Guo et al. (2013), Sanghvi et al. (2014)
		Proteases	*Bacillus* sp., *Pseudoaltermonas* sp., *Halobacillus* spp.		Delgado‐García et al. (2019), Chen et al. (2020)

Cold‐adapted or psychrophilic enzymes are enzymes that can effectively catalyze reactions below 20°C (68°F) and are considered novel food enzymes (Chánique et al. 2023). The cold‐active enzyme has tremendous scope in the food industry. With cold‐active enzymes, cost‐effectiveness is achieved as they can meet activation energy requirements with reduced enzyme quantities (Liu et al. 2023). Moreover, they operate proficiently without additional thermal aid, and their susceptibility to thermal changes enables precise inactivation with minimal heat (Liu et al. 2023). These unique attributes have led to their extensive utilization across the food industry (Javed and Qazi 2016). For example, cold‐active β‐galactosidase is used in the dairy industry to reduce lactose content during milk processing, thereby addressing global concerns related to lactose intolerance (Kuddus 2018).

Cold‐active proteases have extensive applications in food processing for various functional roles, including beer production and speeding up the aging process of cheese. Proteases are crucial in food processing, as they are responsible for tenderizing meat, curdling milk, clarifying beverages, and producing flavors. Protein hydrolysates are also made using cold‐active protease and added to foods like baby formula (Fleischer et al. 2016). Additionally, to improve the mouthfeel and taste of meat products, a unique cold‐active serine protease that was derived from *Chryseobacterium* sp. helped to tenderize meat during refrigeration (Mageswari et al. 2017). Microorganisms adapted to cold environments are promising candidates as a source of cold‐active proteases, with isolation efforts concentrated in frigid regions.

Microbial transglutaminase (MTGase) is commonly applied in food systems to modify protein functionality, hence improving the texture, appearance, firmness, and storage stability of various foods, including meats, fish, and dairy‐based items; protein‐based biodegradable films; and baked goods. In their study, Uran and Yilmaz (2017) examined the quality attributes of chicken burgers made using MTGase. They observed that the burger texture was enhanced with higher enzyme doses when compared to untreated controls (Uran and Yilmaz 2017). In another study, Prakasan et al. (2015) evaluated several approaches that have been proposed for producing natural cheese with MTGase, including adding MTGase to the raw milk prior to pasteurization, which deactivates the enzyme (Prakasan et al. 2015). According to studies, the presence of MTGase in food products may have an impact on the prevalence of celiac disease. Because MTGase can deamidate gluten, it mimics the activity of endogenous tissue TGase, which catalyzes the deamidation of glutamine and is involved in the pathophysiology of celiac disease (Aaron and Torsten 2019). Therefore, there is an increasing safety issue regarding the utilization of MTGase in the production of gluten‐free pastry items.

In the modern food‐processing industry, pectinase enzymes play a crucial role. These enzymes facilitate the decomposition of pectin, an essential carbohydrate found in plants, and are employed in several applications, including the processing of fruit juice (Hamid et al. 2022). Lately, there has been an increasing tendency in the food industry to substitute high‐temperature procedures with low‐temperature procedures, and cold‐active pectinases show great potential here (Moharram et al. 2022). For example, after processing, fresh juice contains suspended pectin, which increases viscosity and haziness and makes filtration difficult. Mesophilic pectinases are commonly used to remove suspended pectin; however, they require ambient or higher temperatures, which increases the danger of contamination and loss of volatile aromatic substances. To address these difficulties, cold‐active pectinases are used (Nakagawa et al. 2004). Another use for cold‐active pectinases is in winemaking, where low‐temperature fermentation is essential for preserving grape flavors. Traditional mesophilic pectinases are less effective at low temperatures and require larger concentrations to achieve excellent results. The use of psychrophilic pectinases addresses this issue, potentially lowering manufacturing costs while improving wine sensory attributes (Adapa et al. 2014). Several other enzymes, such as proteases, amylases, and xylanases, can aid in minimizing dough fermentation time while also retaining fragrances and moisture levels in baked goods (Kuddus 2019b).

Hence, it can be inferred that cold‐active enzymes have a significant potential to make substantial contributions in the field of food biotechnology. To meet the demands of cold‐active enzymes by food processors, different molecular methods could be developed, such as the metagenomic approach and rDNA technology, to make new cold‐active enzymes better in both quality and quantity.

## 8 Safety of Food‐Grade Enzyme

Food‐grade enzymes are referred to as food additives and are generally accepted as harmless. However, allergic reactions, irritations, and toxicity might be caused by industrial enzymes, which are the main safety concerns for consumers. The risks of skin irritation, allergic reactions (in the respiratory tract), chemical toxicity, and residual microbiological activity are mainly concerning for the health of workers involved in producing and using enzymes in industries (Singh, Kundu, et al. 2019).

On the other hand, the risks of oral toxicity are especially important for consumers who use products containing food enzymes (Ramos and Malcata 2011). However, because food formulations often use low amounts of the enzymes, which, after processing, would have finally been disabled before reaching the customers, allergic reactions are rarely reported by the final consumers of food products containing food‐grade enzymes. Future problems that could be related to the safety of food‐grade enzymes include hazardous substances that result from by‐products during the manufacturing of enzymes. The environment of certain phylogenetically distinct strains utilized in the synthesis of enzymes requires careful consideration, as does the presence of fermentation broth contaminants (Ramos and Malcata 2011). The food industry and regulatory bodies at national and international levels need to collaborate in order to create strategies that will standardize and enforce legislation regarding food enzymes that are considered novel or have unique properties. Regarding food enzyme laws, one of the current concerns is that food‐grade enzymes are regarded as food additives in Canada, Japan, and the USA, but as food‐processing aids in Australia (Griffiths and Borzelleca 2014). However, according to the WHO/FAO joint committee on food additives, food‐grade enzymes can be used as both food additives and processing aids (FAO/WHO 2016). Thus, it is crucial for legislation in every country across the globe to establish clear boundaries for the permissible enzymes and their uses.

Finally, researchers, the food industry, and regulatory agencies should conduct investigations and analyses to understand the function of enzymes as respiratory allergens. It is essential to distinguish between enzymes that are not allergic or only mildly allergenic and those that are strongly allergenic when exposed through the respiratory system.

## 9 Challenges and Future Perspective

Despite the expanding applications of enzyme technology in the food industry, several barriers continue to limit their industrial scalability and efficiency. This section outlines key scientific, economic, and regulatory challenges while highlighting future opportunities that align with the broader goal of sustainable and innovative enzyme use.

While enzymes offer numerous benefits, their application in food production is challenging. These include the high cost of enzyme production, potential allergenicity, and the need for precise control over enzymatic reactions. Since they are selective, effective at milder reaction conditions, safe, palatable, and simple to manage, enzymes are used extensively in the food sector (Ackaah‐Gyasi et al. 2015). Nevertheless, ongoing research and technological advancements are focused on overcoming these hurdles, aiming to render enzyme use more accessible and efficient in food production. In the future, enzymes are expected to play a significant role in meeting the growing demand for healthier and sustainable food items. Future developments in enzyme technology focus on creating more robust and more adaptable enzymes using genetic engineering and protein design. Progress in enzyme immobilization and formulation is anticipated to enhance enzyme stability and reusability, which will contribute to sustainability in food production.

The production of enzymes via traditional methods is laborious, time‐consuming, and generally results in minimal yields (Robinson 2015). Recently, applying the technology of recombinant DNA has eliminated many of the typical issues related to conventional enzyme synthesis, resulting in enhanced qualities and cost efficiency. Through the progress in genetic engineering techniques, it is now easy to transfer and express specific genes that code for desired enzymes in compatible host microorganisms. This allows for the efficient manufacture of certain enzymes on a large scale (Schäfer 2007).

There are other emerging enzyme technologies that will provide new opportunities for utilizing enzymes to improve the structural, aromatic, umami, and textural characteristics of food products. Advanced fermentation methods and downstream processing are examples of the kinds of processes that will help produce pure, industrial‐scale food‐processing enzymes (Rastogi and Bhatia 2019). With the development of bioinformatics, the food industries will be significantly impacted by the in silico design of new enzymes and the enhancement of already‐existing enzymes' catalytic efficiency (Minkiewicz et al. 2016).

However, enzymes made traditionally or by updated methods are limited to relatively particular sorts of reactions that occur naturally. To overcome the inherent limitations of natural enzymes, researchers are exploring synthetic alternatives that offer greater robustness and broader catalytic capabilities. Many synthetic organic compounds that resemble natural enzymes are currently being studied to see if they are suitable for use in food applications, particularly in light of the recent increase in research to create food products to satisfy diverse customer wants. These molecules, referred to as artificial enzymes, function on both synthetic and natural substrates and provide models for the in vitro investigation of reactions that may be challenging to investigate with natural enzymes. Artificial enzymes can be purified and recovered with greater ease and in higher quantities compared to real enzymes. Additionally, they exhibit greater resistance to significant variations in temperature, pH, pressure, organic solvents, and ionic strength. These characteristics make them more cost‐effective and efficient to produce. Therefore, artificial enzymes might have a significant impact on the food industry if they can be used safely and successfully in a variety of food‐related industries.

Hence, additional research is required in the field of enzyme technology to enhance the efficiency of production strains, optimize enzyme synthesis for commercial purposes, and devise novel food‐processing methods to achieve the most cost‐effective product formulation.

## 10 Conclusions

The incorporation of enzymes as food‐processing aids or additives is now an established approach; however, research is ongoing to make this biocatalyst more effective and diversified. Several factors, including industry regulations, technological improvements, and market demand, are driving up the need for enzymes in the global market. However, the demand from consumers for more wholesome, safe, and nutritious food has increased the issues faced by food enzymes. In order to increase process effectiveness, product quality, and environmental sustainability, food enzyme research is therefore focused on creating superior composite enzymes.

Furthermore, the extensive range of food components requires enzymes with improved catalytic efficiency, flexible catalytic conditions, and enhanced tolerance. This is anticipated to result in a growing demand for customized enzymes that are specifically designed to meet the unique requirements of different food ingredients and nutritional needs. A few tactics used in the field are creating new enzymes from nature, enhancing already existing catalytic qualities, expanding the use of specialized enzymes to fulfill new roles, and refining the method for enzyme preparations. Future work will also focus on tailoring and refining enzymes for specific food applications to improve their operational stability, reusability, and effectiveness. Overcoming challenges related to scaling up and commercial adoption, while ensuring alignment with regulatory frameworks and market expectations, will be essential for the successful implementation of enzyme‐based innovations in the food sector. As science and technology continue to advance, the use of food enzymes is expected to become more diverse in the future, thus creating new opportunities and advancements for the food industries.

## Author Contributions

**Fahima Siddikey:** methodology (lead), visualization (lead), writing – original draft (equal). **Md Istiakh Jahan:** methodology (equal), writing – original draft (equal). **Hormoni:** methodology (equal), writing – original draft (equal). **Md Toufik Hasan:** methodology (equal), writing – original draft (equal). **Nusrat Jerin Nishi:** methodology (equal), writing – original draft (equal). **S. M. Kamrul Hasan:** methodology (equal), writing – review and editing (equal). **Nahidur Rahman:** methodology (equal), writing – review and editing (equal). **Md. Azmain Al Faik:** methodology (equal), writing – review and editing (equal). **Mohammad Afzal Hossain:** conceptualization (lead), investigation (lead), methodology (equal), project administration (lead), supervision (lead), validation (lead), visualization (supporting), writing – review and editing (lead).

## Conflicts of Interest

The authors declare no conflicts of interest.

## Data Availability

No data has been utilized in this review that requires mention.
